# Combined Effects of Mediterranean Diet Adherence and Physical Activity on Metabolic Homeostasis and Beta-Cell Function in Male Adolescents

**DOI:** 10.3390/nu18091453

**Published:** 2026-04-30

**Authors:** Karin Herrera-Carrasco, Maria Puche-Juarez, Juan Manuel Toledano, Francisco Manuel Ocaña-Peinado, Julio J. Ochoa, Javier Diaz-Castro, Jorge Moreno-Fernandez

**Affiliations:** 1 Department of Physiology, Faculty of Pharmacy, Campus Universitario de Cartuja, University of Granada, 18071 Granada, Spain; jmtoledano@ugr.es (J.M.T.); jjoh@ugr.es (J.J.O.); javierdc@ugr.es (J.D.-C.); jorgemf@ugr.es (J.M.-F.); 2 Institute of Nutrition and Food Technology “Jose Mataix Verdú”, University of Granada, 18071 Granada, Spain; 3 Nutrition and Food Sciences Ph.D. Program, University of Granada, 18071 Granada, Spain; 4 Department of Statistics and Operative Research, Faculty of Pharmacy, Campus Universitario de Cartuja, University of Granada, 18071 Granada, Spain; fmocan@ugr.es; 5 Instituto de Investigación Biosanitaria (IBS), 18016 Granada, Spain

**Keywords:** Mediterranean diet, physical activity, insulin resistance, beta-cell function, adolescent, SPINA indices, HOMA-IR, cardiometabolic risk

## Abstract

Background/Objectives: Adolescence is a critical developmental period during which dietary quality and physical activity (PA) may influence insulin sensitivity and pancreatic β-cell function. This observational cohort study investigated how adherence to the Mediterranean diet (MedDiet) and participation in structured physical activity (PA) relate to metabolic changes over six months in Spanish male adolescents. Methods: A total of 78 participants (median age 11 years; IQR 10–12) were followed in a school-based study (2020–2021) and categorized by MedDiet adherence using the KIDMED index into medium (M) and high (H) groups. Metabolic health was assessed at baseline (T1) and after six months (T2) using lipid profiles, glucose, insulin, and several indirect indices of insulin resistance and β-cell function, including HOMA-IR, QUICKI, and SPINA indices. Statistical analyses included correlations and adjusted linear models, with false discovery rate correction applied. Results: At baseline, higher MedDiet adherence was associated with lower fasting insulin and improved insulin resistance markers (*p* ≤ 0.002). Over six months, adolescents with high adherence showed more favorable changes in insulin sensitivity (fasting insulin, HOMA-IR, QUICKI) and β-cell function (SPINA indices), with results remaining significant after correction (all pFDR < 0.05). LDL cholesterol levels also improved more markedly in participants combining high MedDiet adherence with structured PA (pFDR < 0.001). In contrast, triglycerides and TG-related indices increased across all groups, without differences between them (pFDR < 0.001). Conclusions: High MedDiet adherence combined with structured PA was associated with more favorable trajectories in insulin sensitivity, attenuated β-cell secretory demand, and a more favorable LDL-c profile. These findings support integrated lifestyle approaches for early cardiometabolic prevention in male adolescence.

## 1. Introduction

Puberty is characterized by profound endocrine and body composition changes that are intrinsically associated with a transient reduction in insulin sensitivity, particularly during mid-puberty [1]. This physiological insulin resistance necessitates a compensatory increase in pancreatic β-cell secretion to maintain glycemic homeostasis [2]. When this adaptive balance is disrupted, unfavorable metabolic trajectories may emerge, increasing both short- and long-term cardiometabolic risk [3]. Understanding modifiable lifestyle factors that may be associated with this balance during adolescence therefore has considerable public health relevance.

Within this framework, dietary quality has been recognized as a potential modulator of metabolic health trajectories. The Mediterranean diet (MedDiet) has been consistently associated with improvements in lipid profile, glycemic control, low-grade systemic inflammation, and cardiometabolic disease risk in adult populations [4]. This dietary pattern is characterized by a high intake of fruits, vegetables, legumes, whole grains, nuts, and olive oil as the primary fat source; moderate consumption of fish and dairy products; and low intake of processed meats and ultra-processed foods [5,6]. Evidence regarding its metabolic associations in adolescent populations remains more limited and heterogeneous.

In adolescents, greater adherence to the MedDiet has been associated with a lower prevalence of obesity, more favorable cardiometabolic profiles, and improved diet quality [7,8]. Adherence is commonly assessed using the KIDMED index, a validated and reproducible tool specifically designed for younger populations, which has demonstrated consistent associations with metabolic health and cardiovascular risk indicators [9].

Traditionally, insulin resistance and β-cell function have been assessed using indices such as HOMA-IR and HOMA-β [10]. More recently, mathematical models based on the Structure–Parameter–Inference Approach (SPINA) have enabled a more detailed characterization of the glucose–insulin axis, providing differentiated estimates of basal β-cell secretory capacity (SPINA-Gβ), insulin receptor sensitivity (SPINA-GR), and an integrated disposition index (SPINA-DI), thereby offering a more mechanistically granular assessment of glucose homeostasis [11].

Much of the available evidence derives from cross-sectional studies, which limits the ability to evaluate temporal associations and direction of effects [12]. Intraindividual delta (Δ) analyses reflecting within-person changes over time represent a methodologically more appropriate approach in observational longitudinal research [13,14]. Nevertheless, conclusions from such designs must remain strictly associative.

In addition to diet, physical activity is hypothesized to play a fundamental role in the regulation of energy balance and cardiometabolic health [15]. Regular exercise during adolescence has been associated with improvements in insulin sensitivity, lipid profile, body composition, and cardiovascular health [16]. Despite the biological plausibility of combined effects, the literature on the joint association of dietary quality and structured physical activity in adolescent metabolic trajectories remains limited [17].

In Spain, a progressive departure from the traditional MedDiet has been documented among pediatric and adolescent populations, underscoring the public health relevance of assessing real-world dietary patterns in this demographic [18]. Crucially, the endocrine context of early male adolescence—characterized by rising androgen concentrations, physiological insulin resistance peaking at mid-puberty, and substantial inter-individual heterogeneity in pubertal tempo—introduces biological complexity that may modify or confound lifestyle–metabolism associations in ways not observed in adult cohorts [19].

Against this background, the aim of the present study was to examine the prospective associations between MedDiet adherence, participation in a structured PA program, and intraindividual changes (Δ) in a comprehensive panel of metabolic markers—including conventional glycemic and lipid indices, advanced SPINA-Carb structural parameters of β-cell function and insulin receptor sensitivity, and hepatic biomarkers—in a naturalistic cohort of Spanish male adolescents, using FDR-corrected mixed-model analyses adjusted for body composition and habitual physical activity. We hypothesized that high MedDiet adherence would be associated with lower insulin resistance indices and more favorable β-cell secretory trajectories, that the combination of high adherence and structured exercise would amplify these associations, and that certain biomarker associations would diverge from adult-derived expectations due to the specific endocrine milieu of pubertal development.

## 2. Materials and Methods

### 2.1. Study Design

This prospective naturalistic cohort study included a 6-month follow-up conducted during the 2020–2021 academic year (January–June 2021). The initial sample comprised 103 school-aged male adolescents enrolled at a public school in Granada, Spain. Of these, 25 were excluded due to incomplete biochemical records (*n* = 12), incomplete dietary assessments (*n* = 8), or failure to attend follow-up (*n* = 5), yielding a final analytic sample of 78 participants (Supplementary Figure S1).

Group allocation was naturalistic rather than randomized: participants self-selected into a pre-existing structured PA program (Exercise group (EX), *n* = 32) based on voluntary enrollment, whereas those following standard school curriculum comprised the MedDiet group (MD, *n* = 46). This observational design precludes causal inference; all results must be interpreted as associative. Baseline comparability between groups was confirmed for all measured variables, detailed results and group characteristics are presented in Section 3.1. However, unmeasured confounders—including socioeconomic status, parental dietary practices, and motivational factors—were not assessed. The absence of a randomized controlled design and a formal sedentary control arm represent key structural limitations. Propensity score matching was not performed given the small sample size; baseline covariate adjustment was incorporated into all statistical models.

Inclusion criteria: male sex; age 8–14 years; school enrollment; absence of chronic metabolic or endocrine disease; no medication affecting glucose or lipid metabolism; written informed consent from parents or guardians. Exclusion criteria: type 1 or type 2 diabetes mellitus; insulin or oral hypoglycemic agent use; inflammatory or autoimmune disease; and incomplete baseline or follow-up data.

The full exercise protocol has been described previously [20]. Participants in the Exercise group engaged in a six-month concurrent training program combining moderate-to-vigorous aerobic and resistance exercise. Sessions (initially three per week, each lasting one hour) were structured into a warm-up (10 min), technique and tactical drills (30 min), and game simulation and cool-down (20 min). The Exercise group progressively increased to 100 min per day, five days per week, during the final two months under professional supervision. The MedDiet group maintained the standard physical activity component of the school curriculum throughout.

### 2.2. Anthropometric Assessment

Weight was recorded with a calibrated digital scale (±0.1 kg), and height with a wall-mounted stadiometer (±0.1 cm), each measured in duplicate (third measurement taken if discrepancy exceeded 0.5 kg or 0.5 cm). Body mass index (BMI) was calculated as weight in kilograms divided by the square of height in meters (kg/m^2^). BMI-for-age and height-for-age z-scores (zBMI/A; zH/A) were calculated according to the WHO Growth Reference for school-aged children (5–19 years) [21]. Stunting and thinness, as well as overweight and obesity, were defined based on the standard deviations (SD) established by these international cut-off points.

### 2.3. Biochemical Parameters

Blood was drawn after a minimum 8-h fast. Triglycerides, total and HDL-cholesterol, LDL-cholesterol, AST, ALT, GGT, glucose, insulin, and total bilirubin were analyzed using standard enzymatic and colorimetric techniques on a BS-200 Chemistry Analyzer (Shenzhen Mindray Bio-Medical Electronics Co., Ltd., Shenzhen, China). Each sample was measured in duplicate, and the mean of the two readings was recorded [20].

### 2.4. Adherence to the Mediterranean Diet

Adherence was evaluated using the KIDMED questionnaire (Mediterranean Diet Quality Index for children and adolescents) [22], administered by trained personnel using 24-h recalls at both T1 and T2. No substantive reclassification between adherence categories occurred across time points; the baseline KIDMED score was therefore retained as a stable stratification variable, reflecting habitual dietary pattern. Participants were classified as medium adherence (Medium (M), KIDMED 4–7) or high adherence (High (H), KIDMED ≥ 8) [20].

### 2.5. Physical Activity Measurement

Habitual PA was assessed using the International Physical Activity Questionnaire for Children (IPAQ-C), capturing activity over the preceding seven days. Energy expenditure was expressed in MET-min/week. A standardized MET score (zMETs) was incorporated as a continuous covariate in all models [20].

### 2.6. Metabolic Index

Non-HDL-cholesterol (Non-HDL-c) was calculated as: Non-HDL-c = TC − HDL-c (cutoff ≥ 130 mg/dL for elevated risk). The Castelli Index (TC/HDL-c) was calculated as total cholesterol to HDL-c ratio (cutoff > 3.5 for increased cardiovascular risk) [23]. The triglyceride-glucose index (TyG) was calculated as: TyG = ln [(TG (mg/dL) × G (mg/dL))/2] (cutoff ≤ 8.10) [24]. The TG/HDL-c ratio was calculated as fasting triglycerides (mg/dL) divided by HDL-c (mg/dL) (cutoff < 3.0) [25].

HOMA-β was calculated as: HOMA-β = [20 × I]/[G − 3.5]; and HOMA-IR was calculated as: HOMA-IR = [I × G]/405, where I is fasting insulin (µU/mL) and G is fasting plasma glucose (mg/dL) (cutoff > 2.89, corresponding to the 90th percentile for the Spanish pediatric population) [10,26]. QUICKI was calculated as: QUICKI = 1/[log(I) + log(G)] (values > 0.33 indicative of high insulin sensitivity) [27]. The McAuley index (iMcA) was calculated as: iMcA = exp [2.63 − 0.28 × ln(I) − 0.31 × ln(TG)], where I is fasting insulin (µU/mL) and TG is fasting triglycerides (mmol/L) (values > 6.23 indicative of high insulin sensitivity) [28].

### 2.7. Pancreatic Function and Insulin Sensitivity Indices (SPINA-Carb Indices)

β-cell function and insulin sensitivity were estimated using SPINA-Carb indices calculated from fasting glucose and insulin concentrations [29]. Glucose was converted from mg/dL to mmol/L, and insulin from µIU/mL to pmol/L or nmol/L as required. SPINA-Gβ (basal pancreatic β-cell secretory capacity) was calculated as: SPINA-Gβ = [I × (7 + G)]/(58.8 × G), where I is fasting insulin (pmol/L) and G is fasting glucose (mmol/L). SPINA-GR (insulin receptor sensitivity) was calculated as: SPINA-GR = [50 × (1.6 + I′) × (7 + G)]/(I′ × G), where I′ is fasting insulin (nmol/L). SPINA-DI (disposition index) was obtained as the product of SPINA-Gβ and SPINA-GR. Given the absence of validated clinical cut-off points for adolescent populations, all SPINA indices were analyzed as continuous variables.

### 2.8. Statistical Analysis

Continuous data are reported as mean ± SD or median (IQR). Normality was assessed using the Shapiro–Wilk test (subgroup samples < 50) and the Kolmogorov–Smirnov test (full sample) as a sensitivity check. For categorical variables with cell frequencies below five, adjacent nutritional status categories were collapsed (overweight, obesity, and severe obesity merged) to ensure adequate cell frequencies [30]; individual categories are reported in Table 1 for descriptive transparency.

Spearman rank correlations (ρ) with 95% bootstrap confidence intervals assessed cross-sectional and prospective associations between KIDMED and metabolic variables. Three-way LMMs (REML) included Time (T1, T2), Group (MedDiet, MD; Exercise, EX), and Adherence (Medium, M; High, H) as fixed factors, random intercepts for participant ID, and Satterthwaite-approximated degrees of freedom. Two-way GLM-ANCOVAs (Type III SS) on Δ and Δ% values used Group and Adherence as fixed factors. All models were adjusted for zBMI/A and zMETs. Categorical comparisons used Mann–Whitney U, Pearson χ^2^, or Fisher’s exact test. Post hoc pairwise comparisons used Bonferroni correction.

FDR (Benjamini–Hochberg) was applied within five biologically defined outcome families: (1) glycemic control and insulin sensitivity (Insulin, HOMA-IR, HOMA-β, QUICKI, iMcA); (2) SPINA-Carb indices (SPINA-Gβ, SPINA-GR, SPINA-DI); (3) lipid profile (Triglycerides, Total Cholesterol, LDL-c, HDL-c, Non-HDL-c); (4) cardiometabolic risk indices (TyG, TG/HDL-c, TC/HDL-c); and (5) hepatic biomarkers (Total Bilirubin, AST, ALT, GGT). Nuisance covariates (zBMI/A, zMETs) were excluded from FDR correction. Findings were considered statistically robust only when the FDR-adjusted *p* < 0.05. Unadjusted *p*-values are in Table 2; FDR-adjusted values in Supplementary Table S5.

A post hoc sensitivity analysis (G*Power v3.1.9.6, Düsseldorf, Germany) confirmed that with α = 0.05 and 80% power, the study was sensitive to detect moderate effect sizes (d ≥ 0.65); QUICKI yielded d = 0.68, exceeding this threshold. Analyses were performed in Jamovi v2.7.26.0 (The jamovi project, Sydney, Australia), including the GAMLj v.3.6.5 (jamovi module, https://gamlj.github.io/) and R module v4.5 (R Core Team, Vienna, Austria). Additionally, IBM^®^ SPSS^®^ Statistics v28.0.1.0 (IBM Corp., Armonk, NY, USA) and GraphPad Prism^®^ v10.2.0 (GraphPad Software, Boston, MA, USA) [31,32,33,34].

## 3. Results

### 3.1. Sample Characterization

The final analytic sample comprised 78 male adolescents (MedDiet, *n* = 46; Exercise, *n* = 32; median age 11 years, IQR 10–12). Baseline characteristics are summarized in Table 1. Most participants were classified as normal weight (53.8%); nutritional status distributions did not differ between groups (χ^2^ *p* = 0.068). The median KIDMED score was 7 (IQR 6–8), with no between-group difference (U *p* = 0.135), and adherence strata were equivalently distributed across groups (59.0% Medium vs. 41.0% High; Fisher’s exact *p* = 0.683). Habitual physical activity did not differ between groups at baseline (*p* = 0.115). No statistically significant between-group differences were observed for any variable (all *p* > 0.05), confirming pre-study equivalence.

### 3.2. Baseline Associations Between KIDMED Score and Metabolic Parameters

**Figure 1:**
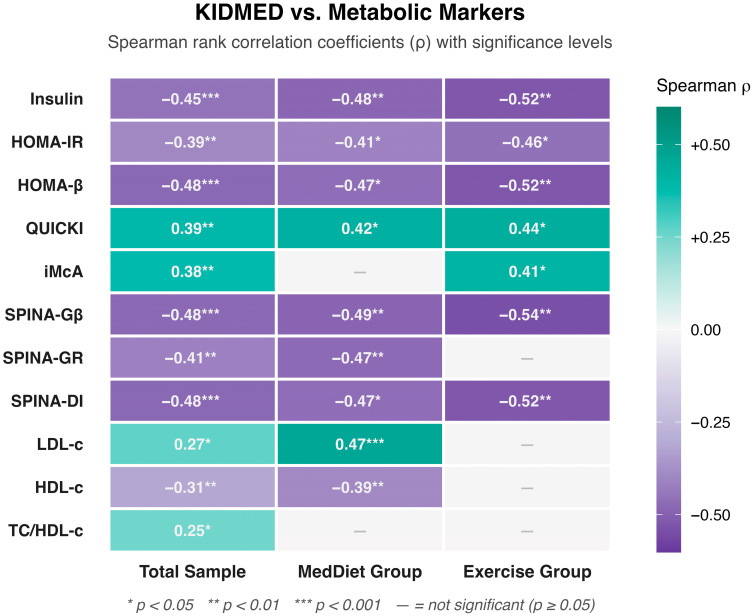
**Relationship between Mediterranean Diet Adherence and Metabolic Health Markers: Correlation Analysis of Lipid Profile, Glucose Metabolism, and SPINA Indices.** Spearman’s rank correlation coefficients (ρ) between baseline KIDMED score and key metabolic parameters, displayed as a heatmap stratified by sample: Total Sample (**left**), MedDiet group (**center**), and Exercise group (**right**). Color intensity reflects the magnitude of ρ, with teal indicating positive and purple indicating negative associations; grey cells (—) denote non-significant correlations (*p* ≥ 0.05). Asterisks indicate significance thresholds: * *p* < 0.05, ** *p* < 0.01, *** *p* < 0.001. Full Spearman correlation matrices including 95% bootstrap confidence intervals are provided in Supplementary Table S1.

The statistically significant correlations between the KIDMED score and metabolic parameters for the total sample and both study groups are shown in Figure 1. At baseline, KIDMED score was inversely and significantly associated with fasting insulin (ρ = −0.449, *p* < 0.001), HOMA-IR (ρ = −0.393, *p* = 0.003), and HOMA-β (ρ = −0.479, *p* < 0.001) across the full sample. These associations were directionally consistent across both the MedDiet and Exercise subgroups (all *p* ≤ 0.027). All three SPINA-Carb indices were inversely associated with KIDMED: SPINA-Gβ (ρ = −0.477, *p* < 0.001), SPINA-GR (ρ = −0.415, *p* < 0.001), and SPINA-DI (ρ = −0.478, *p* < 0.001).

Among lipid variables, LDL-cholesterol showed a positive cross-sectional association with KIDMED in the full sample (ρ = 0.273, *p* = 0.020), driven principally by the MedDiet subgroup (ρ = 0.467, *p* < 0.001), while HDL-cholesterol was inversely associated (ρ = −0.313, *p* = 0.007). The TC/HDL-c ratio was positively associated with KIDMED (ρ = 0.250, *p* = 0.033). Regarding prospective associations between baseline KIDMED and six-month changes (Δ), the most consistent signal emerged for total bilirubin (ρ = −0.594, *p* < 0.001), LDL-cholesterol (ρ = −0.425, *p* = 0.014), and HDL-cholesterol (ρ = −0.434, *p* = 0.010). Full Spearman matrices by group are presented in Supplementary Table S1.

Having established the cross-sectional and prospective associations between baseline dietary adherence and metabolic markers, the following sections present the longitudinal findings from the linear mixed models, organized by metabolic domain.

### 3.3. Glycemic Control, Insulin Sensitivity, and β-Cell Function

Prospective changes in glycemic control, insulin sensitivity and β-cell function were analyzed using linear mixed models (LMM) with random intercepts and general linear models (GLM) on absolute (Δ) and percentage (Δ%) change scores. All models were adjusted for zBMI/A and zMETs. Unadjusted *p*-values for fixed effects are reported in Table 2; FDR-adjusted *p*-values (Benjamini–Hochberg) within biologically defined outcome families and covariate-adjusted estimated marginal means are provided in Supplementary Tables S5 and S4, respectively. The relevant Bonferroni-corrected post hoc comparisons are reported in Table 3.

Fasting plasma glucose remained stable over the six-month period, with no effects of Time, Group, Adherence, or their interactions surviving FDR correction (all pFDR > 0.05).

**Figure 2:**
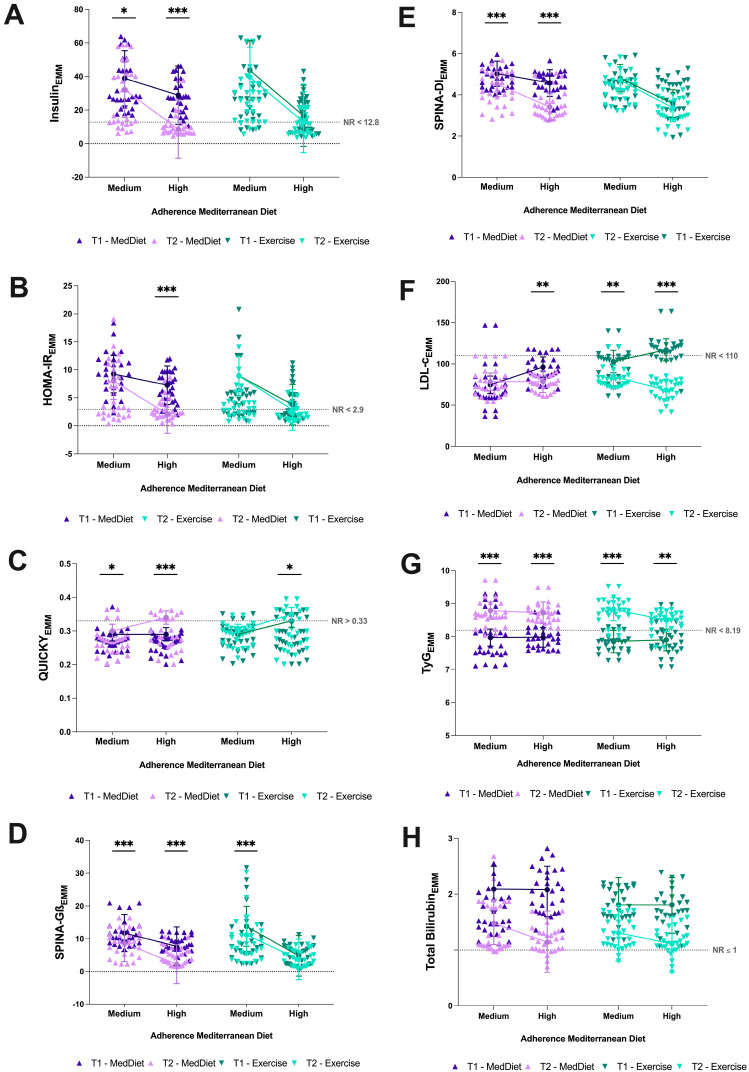
**Six-Month Trajectories in Key Metabolic Markers by Intervention Group and Mediterranean Diet Adherence Level.** Estimated marginal means [± 95% CI] at baseline (T1) and six-month follow-up (T2), stratified by intervention group (MedDiet: purple tones; Exercise: teal tones) and dietary adherence level (Medium: KIDMED 4–7; High: KIDMED ≥ 8), for: (**A**) fasting serum insulin (µUI/mL); (**B**) HOMA-IR; (**C**) QUICKI; (**D**) SPINA-Gβ (pmol/s); (**E**) SPINA-DI; (**F**) LDL-cholesterol (mg/dL); (**G**) triglyceride–glucose index (TyG); and (**H**) total bilirubin (mg/dL). Individual observations are jittered to display the underlying sample distribution (*n* = 78). Dashed horizontal lines indicate age-appropriate pediatric reference ranges (NR). Statistical significance of within-subgroup change from T1 to T2 was assessed by Bonferroni-corrected simple effects from the linear mixed model: * *p* < 0.05, ** *p* < 0.01, *** *p* < 0.001.

**Figure 3:**
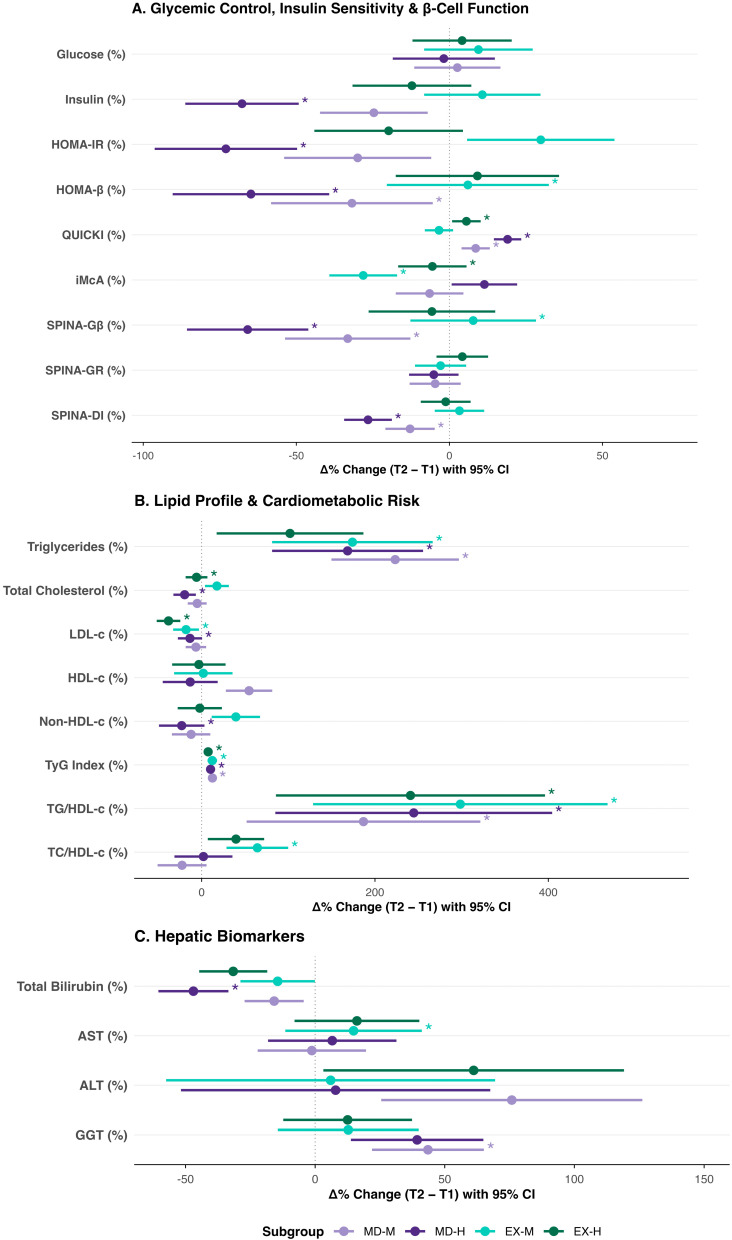
**Intraindividual Percentage Changes (Δ%) in Cardiometabolic and Hepatic Biomarkers by Intervention and Mediterranean Diet Adherence.** Standardized mean percentage changes from baseline (T1) to six-month follow-up (T2) with 95% confidence intervals, stratified by intervention group and dietary adherence. (**A**) Glycemic control, insulin sensitivity, and β-cell function indices; (**B**) Lipid profile and cardiometabolic risk indices; and (**C**) Hepatic biomarkers. Subgroups: MD-M (light purple), MD-H (dark purple), EX-M (light teal), and EX-H (dark teal). Asterisks indicate statistically significant within-subgroup change from baseline (Bonferroni-corrected post hoc tests, *p* < 0.05). Δ%, normalized percentage change [(T2 − T1)/T1 × 100]. The dotted vertical line represents the null value (0), indicating no change from baseline.

Fasting insulin declined over time (Time pFDR < 0.001), with adherence-differentiated trajectories (Adherence pFDR = 0.031; Time × Group pFDR = 0.004). The most pronounced reduction was observed in the MD-H subgroup (from 28.86 to 8.63 µUI/mL); Exercise subgroups showed comparatively attenuated declines. Group effects on both Δ and Δ% survived FDR correction (pFDR = 0.025 and pFDR < 0.001, respectively), as did the Adherence effect on Δ% (pFDR = 0.002). The Time × Group × Adherence interaction did not survive FDR correction (pcrude = 0.022; pFDR = 0.058) and is treated as exploratory (Figure 2A and Figure 3A).

HOMA-IR declined significantly over time (pFDR = 0.004), with significant Adherence (pFDR = 0.014) and Time × Group (pFDR = 0.042) effects. Simple effects confirmed that the reduction was statistically significant only within the MD-H subgroup; Exercise subgroups showed stable HOMA-IR trajectories throughout. Group and Adherence effects on Δ% both survived FDR correction (both pFDR < 0.001) (Figure 2B).

HOMA-β showed a significant Time effect (pFDR < 0.001) and a Group effect on Δ% surviving FDR correction (pFDR = 0.002). The Adherence main effect in the LMM did not survive FDR correction (pFDR = 0.075) and remains exploratory; both MedDiet subgroups showed declines, whereas Exercise subgroups were comparatively stable (Figure 3A).

QUICKI exhibited the most pervasive pattern of FDR-robust associations. The LMM identified significant main effects of Time (pFDR < 0.001) and Adherence (pFDR = 0.004), with significant Time × Group (pFDR = 0.002) and Time × Adherence (pFDR < 0.001) interactions. Group and Adherence effects on both Δ and Δ% survived FDR correction (all pFDR < 0.001). Improvements were confirmed in the MD-H, MD-M, and EX-H subgroups; EX-M remained stable (Figure 2C and Figure 3A).

The McAuley index (iMcA) showed significant LMM effects of Time (pFDR < 0.001), Adherence (pFDR = 0.013), Time × Group (pFDR < 0.001), and Time × Adherence (pFDR < 0.001). Group and Adherence effects on Δ and Δ% survived FDR correction (all pFDR ≤ 0.011). Trajectories diverged markedly: EX-H declined from its highest baseline value, MD-H showed a modest increase, and EX-M showed the steepest decline, while MD-M showed a more modest reduction (Figure 3A).

The insulin sensitivity indices examined in the preceding section reflect surrogate estimates of the glucose–insulin relationship at the whole-body level. To characterize the underlying β-cell secretory and receptor-level contributions more specifically, the following section presents findings from the SPINA-Carb structural indices, which extend the resolution of the conventional HOMA-based framework.

### 3.4. SPINA-Carb Indices: β-Cell Secretory Capacity and Disposition Index

The SPINA-Carb indices provided a complementary characterization of the glucose–insulin axis (Table 2 and Table 3). Given the absence of validated adolescent cut-off points, all three indices were analyzed as continuous variables (marginal R^2^ and conditional R^2^ values are reported in Supplementary Tables S2 and S3)

SPINA-Gβ declined significantly over the six-month period (pFDR < 0.001), with a significant Time × Group interaction (pFDR = 0.017) and a significant three-way Time × Group × Adherence interaction (pFDR = 0.020). The most pronounced decline occurred in the MD-H subgroup (7.71 → 2.25 pmol/s); meanwhile EX-H showed an attenuated response, while the two MD subgroups showed intermediate, directionally parallel reductions. The Adherence main effect did not survive FDR correction (pFDR = 0.054) and is thus treated as exploratory. However, the Group effect on Δ% survived FDR correction (pFDR < 0.001) (Figure 2D and Figure 3A).

SPINA-GR showed a modest main effect of Time that did not survive FDR correction (pcrude = 0.017; pFDR = 0.054). Modest declines were observed across all subgroups, without significant group- or adherence-differentiated patterning (Figure 3A).

SPINA-DI showed significant main effects of Time (pFDR < 0.001) and Adherence (pFDR = 0.011), with significant Time × Group (pFDR < 0.001) and Time × Adherence (pFDR = 0.020) interactions. Group and Adherence effects on Δ survived FDR correction (pFDR < 0.001 and pFDR = 0.031, respectively). The largest reduction was observed in the MD-H subgroup (4.58 → 3.41), whereas EX-H maintained a comparatively stable trajectory; MD-M and EX-M showed intermediate declines (Figure 2E and Figure 3A).

Over the six-month follow-up, lipid trajectories diverged across adherence and intervention strata (Table 2 and Table 3; Supplementary Tables S2 and S3). Whereas triglyceride-based indices rose universally, cholesterol profiles showed adherence- and group-specific modulation.

### 3.5. Lipid Profile

Triglycerides increased across all subgroups (Time pFDR < 0.001). The three-way Time × Group × Adherence interaction reached crude significance (*p* = 0.031) but did not survive FDR correction (pFDR = 0.096) and is therefore treated as exploratory. Notwithstanding the non-significant interaction after FDR correction, the magnitude of increase was directionally attenuated in the EX-H subgroup relative to the other three strata.

Total cholesterol showed a significant effect of Time (pFDR = 0.016) and a Time × Adherence interaction (pFDR = 0.008). Declines were observed in both high-adherence subgroups, whereas MD-M remained stable and EX-M increased slightly. The Adherence effect on both absolute change (Δ, pFDR = 0.019) and percentage change (Δ%, pFDR = 0.016) survived FDR correction (Figure 3B).

LDL-cholesterol exhibited the most robust pattern of differential change. The LMM identified significant effects of Time (pFDR < 0.001), Time × Group (pFDR < 0.001), and Time × Adherence (pFDR < 0.001). Declines were confirmed in three subgroups: MD-H (96.1 → 78.4 mg/dL), EX-M (102.9 → 84.7 mg/dL), and EX-H (117.1 → 68.3 mg/dL); MD-M remained stable. Bonferroni-corrected post hoc comparisons confirmed that the reduction in EX-H substantially exceeded that of all other subgroups (−48.77 mg/dL, *p* < 0.001), while MD-H (−17.69 mg/dL, *p* = 0.004) and EX-M (−18.15 mg/dL, *p* = 0.002) also showed significant within-group declines. In the GLM, both Group (pFDR = 0.032) and Adherence (pFDR = 0.011) effects on absolute change survived FDR correction (Figure 2F).

HDL-cholesterol did not show a significant main effect of Time (pFDR = 0.477). Adherence-divergent trajectories were observed within the MedDiet group—MD-M increased while MD-H declined—while both Exercise subgroups decreased. The Group × Adherence interaction on percentage change reached FDR significance in the GLM (pFDR = 0.043; Figure 3B).

Non-HDL-cholesterol showed crude Time (*p* = 0.016) and Time × Adherence (*p* = 0.022) effects; neither survived FDR correction (pFDR = 0.069 and pFDR = 0.079, respectively). Directionally, the MD-H strata and MD-M declined, while EX-M increased.

Collectively, LDL-cholesterol exhibited the most pronounced adherence-by-intervention interaction, with the EX-H subgroup showing the largest absolute decline. By contrast, triglyceride-derived indices increased across all strata, indicating a uniform pubertal trajectory resistant to differential modulation over this six-month interval.

### 3.6. Cardiometabolic Risk Indices

The triglyceride–glucose index (TyG) increased significantly over time (pFDR < 0.001) across all four subgroups, with no Group or Adherence interaction surviving FDR correction. The estimated marginal means rising from 7.97 to 8.79 in MD-M, from 7.98 to 8.72 in MD-H, from 7.85 to 8.83 in EX-M, and from 7.90 to 8.50 in EX-H. This universal rise was consistent across the entire sample regardless of intervention assignment or dietary adherence level (Figure 2G and Figure 3B; Table 3 and Table S4).

The TG/HDL-c ratio also rose over time (pFDR < 0.001) across all subgroups. The three-way interaction in the LMM did not survive FDR correction (crude *p* = 0.008, pFDR = 0.050). However, the Group × Adherence interaction on absolute change (Δ) was FDR-robust in the GLM (pFDR = 0.029; Figure 3B; Table S5).

The TC/HDL-c ratio showed a significant Time × Group interaction (pFDR = 0.008). The ratio decreased in the MedDiet arm (MD-M: 4.90 → 4.07; MD-H: 5.28 → 5.60) and increased in the Exercise arm across both adherence strata (EX-M: 4.58 to 6.68; EX-H: 5.45 to 6.96). The Group effect on both absolute and percentage change survived FDR correction (both pFDR = 0.016; Figure 3B; Table 3).

Within the cardiometabolic risk domain, the most prominent adherence-by-intervention signal emerged for LDL-cholesterol, while triglyceride-derived indices followed a pubertal trajectory that was largely independent of lifestyle strata. The following section examines how these lipid trajectories were accompanied by changes in hepatic biomarkers.

### 3.7. Hepatic Biomarkers

Total bilirubin declined over the follow-up period (Time pFDR = 0.002). In the GLM on Δ, significant Adherence (pFDR = 0.004) and Group × Adherence (pFDR = 0.017) effects were observed. The Adherence effect on Δ% also survived FDR correction (pFDR = 0.004). EX-H showed the largest bilirubin decline; Bonferroni-corrected post hoc analyses confirmed this subgroup declined significantly more than MD-M (*p* = 0.017) and EX-M (*p* = 0.001). All values remained within the physiologically normal pediatric range at both time points. The Time × Group, Time × Adherence, and Time × Group × Adherence interactions did not survive FDR correction in the LMM (all pFDR > 0.05; Figure 2H and Figure 3C; Table 3).

GGT increased modestly over time (pFDR = 0.013), with the rise driven predominantly by the MD-M subgroup; the Group × Adherence interaction on Δ did not survive FDR correction (pFDR = 0.236).

AST showed no FDR-robust main effects or interactions (all pFDR > 0.05). The three-way Time × Group × Adherence interaction reached significance at the crude level (pcrude = 0.035; pFDR = 0.236) and is treated as exploratory; EX-M showed the most prominent within-group decline (−8.1 U/L, *p* = 0.001 in post hoc), while other subgroups remained stable (Table 3).

ALT showed no FDR-robust main effects or interactions (all pFDR > 0.05). The three-way interaction reached significance at the crude level (pcrude = 0.030; pFDR = 0.236) and is treated as exploratory. MD-M showed the most directionally distinct trajectory (increase), in contrast to the overall stability of remaining subgroups (Table 2 and Table S5).

Taken together, these results indicate that high MedDiet adherence is associated with more favorable insulin sensitivity and β-cell secretory efficiency, whereas structured exercise amplifies these effects most distinctly in the lipid domain. The following sections interpret these patterns in the context of pubertal physiology and lifestyle intervention mechanisms.

## 4. Discussion

The present study investigated prospective associations between adherence to the Mediterranean diet (MedDiet), structured physical activity, and intra-individual changes in a comprehensive panel of metabolic markers in male adolescents, using false discovery rate (FDR)-corrected multilevel models. Across the full dataset, the analytical dimensions that consistently remained significant after FDR correction were Group (intervention), Time, and Adherence, along with their pairwise and three-way interactions on intra-individual Δ and Δ%. This pattern suggests that dietary adherence is a primary determinant of within-person metabolic change during pubertal development, while structured physical activity appears to act as a complementary modifier. Its effects were most evident in the lipid profile and, to a lesser extent, in hepatic biomarkers, particularly in interaction with levels of dietary adherence.

### 4.1. Adherence as the Dominant Metabolic Modulator: Insulin Sensitivity and β-Cell Function

The most consistent and clinically meaningful signal in this dataset is the FDR-robust divergence in insulin sensitivity trajectories according to MedDiet adherence, independent of intervention group. Participants classified as MD-H exhibited a substantially lower insulinemic burden at both time points, with the most pronounced prospective reductions observed in the MD-H subgroup. This pattern is consistent with a stable metabolic baseline established and maintained by high dietary adherence across the developmental window studied, rather than reflecting a transient response to a dietary prescription [35].

The biological plausibility of this association is well grounded in the mechanistic literature. The dietary composition indexed by high KIDMED scores provides a convergent set of bioactive nutrients—monounsaturated fatty acids (MUFAs) from olive oil, dietary fiber from legumes and whole grains, and plant polyphenols from vegetables and nuts—that act synergistically on insulin signaling pathways [36,37]. MUFAs enhance insulin receptor substrate-1 (IRS-1) signaling efficiency through membrane phospholipid remodeling [38]; dietary fiber attenuates postprandial insulinemia by slowing intestinal carbohydrate absorption and modulating glucagon-like peptide-1 (GLP-1) secretion [39]; and polyphenols—including hydroxytyrosol, oleuropein, and quercetin—activate AMPK-dependent pathways that upregulate GLUT4 trafficking in peripheral tissues [40,41]. Collectively, these mechanisms reduce the chronic secretory demand placed on pancreatic β-cells during a developmental period in which physiological workload is intrinsically elevated due to puberty-associated transient insulin resistance [42].

The pervasive QUICKI signal—exhibiting significant Time, Adherence, and interaction effects across both LMM and GLM frameworks—reinforces this interpretation. QUICKI is logarithmically calibrated and particularly sensitive to changes at the lower end of the insulin distribution, precisely the physiological range in which high-adherence subgroups operate [43]. The observation that improvements in QUICKI were confined to the MD-H and MD-M subgroups, while EX-M remained stable, indicates that adherence—rather than exercise exposure per se—is the necessary condition for a favorable insulin sensitivity trajectory in this cohort [44].

The McAuley index (iMcA), which incorporates fasting triglycerides and thereby reflects adipose tissue–mediated insulin sensitivity, exhibited a biologically meaningful divergence between EX-H and MD-H. This dissociation may reflect the acute lipolytic effects of concurrent exercise training, which transiently elevate circulating free fatty acids and fasting triglycerides, thereby temporarily suppressing the iMcA signal independently of improvements in muscular glucose disposal [45,46]. This interpretation remains speculative; hyperinsulinemic–euglycemic clamp studies with concurrent adipokine measurements would be required to test this hypothesis rigorously [47].

The declines in HOMA-β and SPINA-Gβ, most pronounced in MD-H subgroups, are mechanistically consistent with a reduction in the chronic stimulus for compensatory insulin hypersecretion rather than with β-cell secretory failure [48,49]. This interpretation is supported by the concurrent stability of fasting plasma glucose across all subgroups, indicating preserved β-cell adaptive capacity throughout the study period [50]. The reduction in these secretory indices should therefore be interpreted as a signal of metabolic efficiency associated with improved peripheral insulin sensitivity, rather than as evidence of β-cell insufficiency—a distinction relevant to preventing exhaustion-driven secretory decline along the longer-term cardiometabolic trajectory [51,52].

SPINA-DI, the integrated disposition index, exhibited the most complex interaction structure among the indices, with significant Time × Group and Time × Adherence effects surviving FDR correction. The relatively stable SPINA-DI trajectory in EX-H, in contrast to the decline observed in MD-H, is consistent with the hypothesis that concurrent aerobic and resistance training may upregulate insulin-independent glucose disposal pathways in skeletal muscle, thereby partially preserving overall glucoregulatory reserve [53,54]. However, this mechanistic pathway cannot be confirmed from fasting glucose and insulin data alone; clamp-based and molecular studies are required to characterize this effect definitively [55].

The SPINA-Carb framework provides mechanistic depth beyond conventional HOMA-based indices by differentiating between secretory capacity (SPINA-Gβ), receptor sensitivity (SPINA-GR), and their integrated product (SPINA-DI). The observation that SPINA-GR did not show FDR-robust changes, whereas SPINA-Gβ and SPINA-DI did, suggests that adherence-associated improvements in this cohort are primarily driven by a reduction in secretory demand rather than by structural changes in insulin receptor sensitivity—a distinction not captured by HOMA-IR alone [11,29,56].

### 4.2. Preserved Glycemic Homeostasis as a Mechanistically Coherent Reference Point

The stability of fasting plasma glucose throughout the study period, in the absence of any FDR-robust temporal or group effects, should not be interpreted as a null finding. In the context of puberty-associated insulin resistance—in which the β-cell compensates for declining peripheral sensitivity by augmenting insulin output—preserved euglycemia across all subgroups constitutes direct evidence of intact β-cell adaptive capacity [57,58]. The dissociation between stable fasting glucose and markedly divergent insulin sensitivity indices supports the validity of the analytical framework employed, confirming that HOMA-IR, QUICKI, and SPINA-DI capture metabolically informative variation that is not detectable from glucose measurements alone [59]. This observation is consistent with the established understanding that pubertal insulin resistance does not necessarily manifest as overt dysglycemia in otherwise healthy adolescents, thereby reinforcing the utility of sensitive surrogate indices for early cardiometabolic risk characterization at the preclinical stage [60,61].

### 4.3. Interaction of Intervention and Adherence on LDL-Cholesterol: A Synergistic Lipid Signal

LDL-cholesterol trajectories provided the clearest evidence of interaction between intervention type and adherence stratum within the lipid domain. The EX-H combination produced the largest absolute reduction in LDL-c of any subgroup, substantially exceeding the reductions observed in both MD-H and EX-M, which also declined significantly. In contrast, the MD-M subgroup remained stable over the follow-up period.

The biological coherence of this synergistic pattern is supported by converging mechanistic evidence. High MedDiet adherence optimizes the exogenous dietary lipid substrate: dietary fiber enhances bile acid excretion and reduces hepatic LDL receptor downregulation [62]; MUFAs from olive oil selectively reduce circulating apoB-100–containing LDL particles [63]; and the polyphenol-rich dietary matrix attenuates hepatic VLDL synthesis via NF-κB inhibition and PPAR-α activation [64,65]. Structured concurrent exercise independently upregulates skeletal muscle lipoprotein lipase (LPL) activity and enhances LDL receptor expression in hepatocytes, thereby increasing receptor-mediated LDL clearance [66,67,68]. The combination of a diet that attenuates hepatic LDL production with an exercise program that enhances peripheral LDL clearance provides a mechanistically coherent explanation for the synergistic magnitude observed in the EX-H subgroup [69].

The stability observed in MD-M suggests that a threshold of dietary adherence may be required before the exercise-amplification effect is expressed in the lipid domain [70,71]. Whether this threshold is intrinsic to dietary composition or reflects a lower baseline LDL production rate in MD-M participants cannot be determined from the present data. These findings are consistent with recent evidence indicating that the lipid-lowering effects of Mediterranean-style dietary patterns in pediatric populations are attenuated at moderate adherence levels and amplified by concurrent physical activity [72]. They also align with intervention data from adult Mediterranean cohorts demonstrating the greatest LDL-c reductions among participants combining high dietary quality with structured exercise [44,73].

The cross-sectional positive association between KIDMED score and LDL-cholesterol at baseline—which contrasts with the direction typically observed in adult populations—is most plausibly explained by two converging mechanisms. First, rising testosterone concentrations in early male adolescence are a well-documented driver of LDL-c elevation at Tanner stages II–III [74]; if MD-H adolescents were at a more advanced pubertal stage at baseline, this endocrine effect could dominate the cross-sectional lipid signal [75]. Second, the fatty acid composition of a high-quality Mediterranean diet in adolescence—rich in olive oil, legumes, and nuts—may transiently shift LDL particle distribution toward larger, more buoyant particles, a phenomenon distinct from the atherogenic profile associated with small, dense LDL [76]. Importantly, this cross-sectional association reversed prospectively, with higher baseline KIDMED scores predicting greater LDL-c reductions over six months, indicating that the baseline pattern reflects a confounded snapshot rather than a diet-induced atherogenic trajectory.

### 4.4. Total Bilirubin: An Adherence-Modulated Hepatic Signal Warranting Cautious Interpretation

Total bilirubin declined across all subgroups over the follow-up period, with significantly greater reductions observed in high-adherence participants and, specifically, in the EX-H combination. This pattern is reflected in the FDR-robust Adherence and Group × Adherence effects on Δ and Δ% in the GLM analyses. All values remained within the physiologically normal pediatric range at both time points, and the temporal interaction effects in the LMM analyses did not survive FDR correction, thereby qualifying the robustness of this finding. Bilirubin is an endogenous antioxidant that scavenges reactive oxygen species and inhibits lipid peroxidation at physiological concentrations [77]. Epidemiological data in adults indicate inverse associations between circulating bilirubin levels and the incidence of metabolic syndrome and non-alcoholic fatty liver disease [78]. Whether the adherence-differentiated bilirubin dynamics observed in this study reflect true modulation of the heme oxygenase-1 (HO-1)/biliverdin reductase antioxidant axis—potentially mediated by dietary polyphenols and exercise-induced redox signaling—cannot be determined from the present data [79,80]. The concurrent stability of AST, ALT, and GGT across all subgroups—with only a modest increase in GGT in the MD-M subgroup reaching FDR significance—suggests the absence of hepatocellular stress [81]. This supports the interpretation that the observed variation in bilirubin reflects differential modulation of physiological antioxidant dynamics rather than underlying hepatic pathology [82]. Nevertheless, given the observational design, limited sample size, and the fact that the primary LMM temporal interactions for this variable did not survive FDR correction, the bilirubin findings should be considered hypothesis-generating and warrant prospective validation using direct biomarkers of oxidative stress and comprehensive hepatic functional assessment in future studies.

### 4.5. The Pubertal Metabolic Floor: Universal Triglyceride and Cardiometabolic Risk Index Elevation

The universal and undifferentiated increase in fasting triglycerides, TyG index, and TG/HDL-c ratio across all group–adherence combinations—without any Group or Adherence interactions surviving FDR correction—constitutes strong evidence of a pubertal metabolic floor that is largely resistant to lifestyle modification within the time frame studied [83,84,85]. This pattern is consistent with well-documented features of early male pubertal development, including androgen-driven hepatic VLDL assembly, growth hormone–mediated adipocyte lipolysis, and the relative immaturity of peripheral lipoprotein lipase (LPL) activity [74,86,87]. In adolescent males, a progressive increase in fasting triglycerides between Tanner stages I and III has been robustly demonstrated in large population-based studies, with a magnitude substantially exceeding that attributable to dietary or exercise effects in observational designs [88,89].

The TyG index, validated as a surrogate marker of insulin resistance in pediatric populations [24], followed the same universal pubertal trajectory, confirming that triglyceride-based composite risk indices track this hormonal baseline in this age group, largely independent of lifestyle factors [90]. The directionally attenuated triglyceride increase observed in the EX-H subgroup did not survive FDR correction and therefore cannot be interpreted as a statistically robust intervention effect, rather, it should be considered a hypothesis-generating observation for future randomized trials with longer follow-up, larger sample sizes, and formal pubertal staging as a stratification variable.

The Group effect on the TC/HDL-c ratio—which increased exclusively in Exercise subgroups and survived FDR correction—remains incompletely explained by the available data. Whether this finding reflects a differential effect of concurrent training on lipoprotein particle remodeling, a transient exercise-induced shift in HDL composition, or residual dietary differences between groups warrants further investigation using lipoprotein subfractionation methodologies in future studies [91,92,93,94].

### 4.6. Limitations

Several structural limitations constrain the interpretation of these findings. The absence of pubertal staging (Tanner scores) and hormonal measurements (e.g., testosterone and IGF-1) represents the most critical limitation of the study; pubertal stage is a primary confounder for both lipid and insulin-related outcomes, and its omission cannot be readily compensated for in the analytical framework. The exclusively male, single-school sample further limits generalisability to female adolescents and to broader sociocultural contexts. The relatively small sample size (N = 78) may have attenuated between-subgroup contrasts, particularly in analyses involving four-cell stratification. The KIDMED index does not capture food quantity or within-category variability in dietary intake and was administered without formal stratification by pubertal stage. In addition, group allocation was naturalistic rather than randomized, precluding causal inference and introducing the possibility of unmeasured confounding, including socioeconomic status, parental dietary patterns, and motivational factors. Propensity score matching was not performed due to the limited sample size, although baseline covariate adjustment was incorporated into all models. Future studies should incorporate sex-balanced designs, formal pubertal staging, direct hormonal measurements, lipoprotein subfractionation, and validated oxidative stress biomarkers to address these limitations and to rigorously test the mechanistic hypotheses generated by the present findings.

### 4.7. Integrative Perspective and Clinical Relevance

Considered in aggregate, the present findings support a model in which high MedDiet adherence is associated with a primary metabolic anchor during early male adolescent development, establishing a lower insulinemic baseline, facilitating more favorable lipid trajectories, and maintaining physiological hepatic antioxidant dynamics [95]. In this framework, structured physical activity functions as a synergistic amplifier, with its most pronounced effects observed in the LDL-c domain. The SPINA-Carb framework provided additional mechanistic resolution beyond conventional HOMA-based indices, enabling differentiation between trajectories of secretory capacity and receptor sensitivity. The naturalistic, school-based design enhances ecological validity but precludes formal causal inference; accordingly, all observed associations are observational, and bidirectional or reverse causal mechanisms cannot be excluded.

The universal increase in triglyceride-based cardiometabolic indices reflects a pubertal metabolic floor driven by androgen-mediated hepatic lipogenesis, which appears largely resistant to lifestyle modification within the time frame of observation [96]. Collectively, these findings support integrated lifestyle strategies for early cardiometabolic risk prevention in male adolescence [97], while underscoring the need for randomized, pubertally stratified trials to establish causal directionality and to determine whether the synergistic LDL-c effects observed in this study can be replicated under controlled experimental conditions.

## 5. Conclusions

High adherence to the Mediterranean diet, particularly when combined with structured physical exercise, was associated with more favorable metabolic trajectories in male adolescents over a six-month observational period. The statistically robust analytical dimensions were Intervention, Time, and Adherence, along with their interactions on intra-individual Δ and Δ%, and these effects were consistent across insulin sensitivity indices, β-cell function parameters, and LDL-cholesterol trajectories. SPINA-Carb indices provided additional mechanistic granularity beyond HOMA-based estimates by differentiating trajectories of secretory capacity from those of receptor sensitivity. Total bilirubin demonstrated adherence-modulated dynamics within the physiological range, however, this finding is hypothesis-generating and requires prospective validation. The universal increase in triglyceride-based cardiometabolic indices reflects a pubertal metabolic floor driven by androgen-mediated hepatic lipogenesis, which appears largely resistant to lifestyle modification within the observation window. All findings should be interpreted as associative given the naturalistic, observational study design.

## Figures and Tables

**Table 1:** Baseline characteristics of participants by intervention group.

	All (*n* = 78)		MedDiet (*n* = 46)		Exercise (*n* = 32)		*p*-Value	Effect Size
Continuous variables: median (IQR) or mean ± SD as appropriate.								
Age (years)	11	(10–12)	11	(10–12)	11	(10–12)	0.473 U	−0.092 r
Weight (kg)	42.2	(35.2–50.2)	43.1	(38.0–50.2)	38.3	(32.7–48.6)	0.064 U	−0.248 r
Height (m)	1.47	±0.10	1.48	±0.08	1.45	±0.11	0.156 †	0.330 d
zBMI/A	0.64	±1.32	0.94	±1.29	0.20	±1.27	0.072 †	0.420 d
zH/A	−0.07	±0.96	0.09	±0.81	−0.30	±1.12	0.073 †	0.418 d
METs (min per week)	2031.1	±254.80	1993.2	±270.1	2085.7	224.0	0.115 †	−0.367 d
KIDMED score	7	(6–8)	7	(6–8)	7	(6.0–8.8)	0.135 U	0.196 r
Categorical variables: n (%)								
Nutritional status							0.068 χ^2^	0.263 V
Underweight	6	(7.7)	—	—	6	(18.8)		
Normal weight	42	(53.8)	26	(56.5)	16	(50.0)		
Overweight	14	(17.9)	6	(13.0)	8	(25.0)		
Obesity	14	(17.9)	12	(26.1)	2	(6.3)		
Severe obesity	2	(2.6)	2	(4.3)	—	—		
zH/A classification							0.083 χ^2^	0.253 V
Low normal	12	(15.4)	4	(8.7)	8	(25.0)		
Normal	56	(71.8)	38	(82.6)	18	(56.3)		
Tall	8	(10.3)	2	(4.3)	6	(18.8)		
Very tall	2	(2.6)	2	(4.3)	—	—		
KIDMED classification							0.683 f	−0.046 ø
Medium adherence	46	(59.0)	28	(60.9)	18	(56.3)		
High adherence	32	(41.0)	18	(39.1)	14	(43.8)		

Abbreviations: IQR, interquartile range; M, mean; SD, standard deviation; *n*, absolute frequency; %, relative frequency; zBMI/A, body mass index-for-age z-score (WHO reference); zH/A, height-for-age z-score (WHO reference); METs, Metabolic Equivalent of Task; KIDMED, Mediterranean Diet Quality Index for Children and Adolescents. Statistical tests: †, independent samples *t*-test (normal distribution confirmed by Shapiro–Wilk; homogeneity of variance by Levene’s test); U, Mann–Whitney U test (non-normal distribution); χ^2^, Pearson’s chi-squared test; f, Fisher’s exact test. Effect size measures: d, Cohen’s d (continuous variables, independent *t*-test; small ≥ 0.20, medium ≥ 0.50, large ≥ 0.80); r, rank-biserial correlation (Mann–Whitney U; small ≥ 0.10, medium ≥ 0.30, large ≥ 0.50); V, Cramér’s V (chi-squared; small ≥ 0.10, medium ≥ 0.30, large ≥ 0.50); ø, phi coefficient (Fisher’s exact, 2 × 2 tables); all effect sizes are absolute values. No significant baseline differences were observed between groups for any variable (all *p* > 0.05), supporting pre-study equivalence. For categorical comparisons, adjacent categories with cell frequencies below five were collapsed, individual categories are retained in the table above for descriptive transparency (see Section 2.8).

**Table 2:** Summary of statistical effects on metabolic and biochemical outcomes across all models.

Variable	Linear Mixed Model—Fixed Effects									GLM—Absolute Δ					GLM—Percentage Δ%				
	T	G	A	zBMI/A	zMETs	T×G	T×A	G×A	T×G×A	G	A	zBMI/A	zMETs	G×A	G	A	zBMI/A	zMETs	G×A
**Glycemic Control & Insulin Sensitivity**																			
Glucose	0.519	0.735	0.159	0.941	0.761	0.724	0.363	0.563	0.783	0.642	0.355	0.977	0.180	0.826	0.496	0.519	0.458	0.180	0.955
Insulin	**<0.001**	0.913	**0.011**	0.768	0.056	**0.001**	**0.024**	0.531	**0.022**	**0.009**	0.063	0.792	0.283	0.091	**<0.001**	**<0.001**	0.769	0.050	0.268
HOMA-IR	**0.001**	0.771	**0.005**	0.994	**0.036**	**0.016**	**0.033**	0.593	0.174	**0.026**	0.070	0.739	0.166	0.346	**<0.001**	**<0.001**	0.406	**0.009**	0.776
HOMA-β	**<0.001**	0.737	**0.032**	0.479	0.111	0.068	0.686	0.597	0.060	0.216	0.854	0.092	0.538	0.307	**<0.001**	0.228	0.898	0.443	0.163
QUICKI	**<0.001**	0.248	**0.001**	0.836	0.052	**<0.001**	**<0.001**	0.246	0.261	**<0.001**	**<0.001**	0.084	**0.003**	0.959	**<0.001**	**<0.001**	0.109	**0.005**	0.743
iMcA	**<0.001**	0.334	**0.004**	0.950	0.056	**<0.001**	**<0.001**	0.146	0.543	**<0.001**	**<0.001**	**0.026**	0.817	0.793	**0.004**	**<0.001**	0.166	0.517	0.662
**β-cell Function & Insulin Sensitivity—SPINA-Carb Indices**																			
SPINA-Gβ	**<0.001**	0.815	**0.021**	0.547	0.087	**0.004**	0.610	0.590	**0.020**	**0.027**	0.563	0.161	0.989	0.177	**<0.001**	**0.018**	0.559	0.141	0.336
SPINA-GR	**0.017**	0.805	**0.042**	0.474	0.240	0.100	0.255	0.721	0.263	0.516	0.369	0.207	0.209	0.513	0.251	0.394	0.558	0.263	0.353
SPINA-DI	**<0.001**	0.453	**0.002**	0.713	0.067	**<0.001**	**0.005**	0.407	**0.031**	**<0.001**	**0.008**	0.058	0.230	0.345	**<0.001**	**0.018**	0.145	0.257	0.241
**Lipid Profile**																			
Triglycerides	**<0.001**	0.539	0.140	0.664	0.828	0.693	0.096	0.651	**0.031**	0.570	0.143	0.461	0.413	0.112	0.242	0.111	0.247	0.051	0.838
Total Cholesterol	**0.002**	**0.021**	0.271	**0.008**	0.573	0.265	**<0.001**	0.863	0.783	**0.042**	**0.003**	0.797	**0.036**	0.614	**0.015**	**0.002**	0.820	**0.033**	0.470
LDL-c	**<0.001**	0.094	0.374	0.536	0.764	**<0.001**	**<0.001**	0.276	0.412	**0.005**	**0.001**	0.907	0.983	0.544	**0.025**	**0.036**	0.291	0.893	0.322
HDL-c	0.369	0.222	**0.010**	0.109	0.090	0.193	0.059	0.541	0.137	0.224	0.146	0.164	0.938	0.087	0.235	**0.013**	0.549	0.911	**0.043**
Non-HDL-c	**0.016**	**0.042**	0.819	**0.021**	0.986	0.098	**0.022**	0.969	0.303	**0.013**	**0.034**	0.345	0.056	0.182	**0.017**	**0.030**	0.504	0.063	0.225
**Cardiometabolic Risk Indices**																			
TyG Index	**<0.001**	0.555	0.529	0.722	0.771	0.967	0.084	0.687	0.283	0.670	0.148	0.525	0.310	0.430	0.617	0.143	0.488	0.251	0.598
TG/HDL-c	**<0.001**	0.929	0.510	0.844	0.854	0.385	0.666	0.459	**0.008**	0.555	0.847	0.341	0.694	**0.029**	0.550	0.997	0.082	0.285	0.453
TC/HDL-c	**0.009**	0.204	0.209	0.907	0.543	**<0.001**	0.637	0.751	0.140	**0.002**	0.865	0.573	0.179	0.134	**0.002**	10.000	0.597	0.189	0.129
**Hepatic Biomarkers**																			
Total Bilirubin	**<0.001**	0.921	0.116	0.381	0.137	**0.005**	**<0.001**	**0.012**	**<0.001**	0.992	**<0.001**	0.450	**0.006**	**0.002**	0.348	**<0.001**	0.419	**0.003**	0.100
AST	0.187	0.399	0.076	0.521	0.420	0.141	0.106	0.883	**0.035**	0.494	0.345	0.098	**0.044**	0.491	0.361	0.683	0.174	**0.049**	0.782
ALT	0.514	0.911	0.529	0.528	0.389	0.506	0.132	0.158	**0.030**	0.877	0.296	0.096	0.562	**0.047**	0.805	0.813	0.093	0.880	**0.035**
GGT	**0.001**	0.940	0.673	0.703	0.884	0.309	0.178	**0.043**	0.168	0.277	0.380	0.153	0.919	0.162	0.050	0.849	0.374	0.633	0.873

Note. Each cell shows the *p*-value for all fixed effects and interactions in the linear mixed model and general linear models on absolute and percentage change scores. Bold red: *p* < 0.05; grey: *p* ≥ 0.05. All models adjust for zBMI/A (BMI-for-age z-score) and zMETs (standardized physical activity load) as covariates. **LMM:** Linear mixed model (REML) with random intercepts for individual ID. T, Time (T1 vs. T2); G, Group (MedDiet vs. Exercise); A, Adherence (MD-M vs. MD-H); T×G, T×A, G×A, two-way interactions; T×G×A, three-way interaction. R^2^marginal and R^2^conditional per variable in Supplementary Table S2. **GLM:** General linear model (ANCOVA, Type III SS). Δ = absolute intraindividual change (T2 − T1); Δ% = normalized percentage change [(T2 − T1)/T1 × 100]. G×A = Group × Adherence interaction. Full F-statistics, β, η^2^p, ω^2^, and Adj. R^2^ in Supplementary Table S3. Abbreviations: iMcA, McAuley index; TyG, triglyceride–glucose index; TG/HDL-c, triglyceride-to-HDL cholesterol ratio; TC/HDL-c, total cholesterol-to-HDL cholesterol ratio; SPINA-Gβ, β-cell secretory capacity; SPINA-GR, insulin receptor sensitivity; SPINA-DI, disposition index; AST, aspartate aminotransferase; ALT, alanine aminotransferase; GGT, gamma-glutamyl transferase.

**Table 3:** Subgroup-specific metabolic trajectories: verified post hoc and simple effects summary (FDR-robust findings).

Variable (Units)	LMM/GLM Source Term	Subgroup/Contrast	Adjusted Mean (T1 → T2)	Group Difference (Δ or Δ%)	Magnitude of Change	*p* Bonf.	Clinical Interpretation
**Glycemic Control & Insulin Sensitivity**							
**Insulin** **(µIU/mL)**	T×G	MD-H: T1 → T2	28.9 → 8.6	—	↓ 20.2	**<0.001**	Largest fasting insulin reduction; exclusive to MD-H. MD-M showed a smaller significant ↓ (38.9 → 32.1, ↓ 6.8, *p* = 0.015). Both Exercise subgroups were stable (EX-M *p* = 0.154; EX-H *p* = 0.228).
	G(Δ); G(Δ%)	MD-H vs. EX-H	—	18.7	↓ 55.5% vs. ↓ 78.5%	**0.013**	MD-H achieved greater absolute and percentage insulin reduction than both Exercise subgroups.
	A (Δ%)	High vs. Medium (cross-group)	—	~33%	↓	**<0.001**	High adherence independently drove ~33% greater percentage insulin reduction, regardless of intervention.
**HOMA-IR**	T×G	MD-H: T1 → T2	7.26 → 2.14	—	↓ 5.12	**<0.001**	Clinically significant ↓ exclusive to MD-H (approaching pediatric threshold < 2.89). All other subgroups stable.
	G(Δ%); A(Δ%)	MD-H vs. EX-M	—	102.8%	↓	**<0.001**	MD-H exceeded EX-M by >100 percentage points. High adherence (cross-group): ~46% greater %ΔHOMA-IR.
**HOMA-β (%)**	T	MD-H: T1 → T2	100.5 → 26.2	—	↓ 74.3	**<0.001**	Universal temporal ↓ in three subgroups (MD-H ↓ 74.3, MD-M ↓ 50.7, EX-M ↓ 51.6); EX-H stable. Interpreted as attenuation of compensatory β-cell hypersecretion (euglycaemia preserved).
	G(Δ%)	MD-H vs. EX-H	—	74.0%	↓	**0.002**	MD-H markedly outperformed EX-H in %ΔHOMA-β; also exceeded EX-M (↓ 70.8%, *p* = 0.004).
**QUICKI**	T×G; T×A	MD-H: T1 → T2	0.291 → 0.338	—	↑ 0.047	**<0.001**	↑ in three subgroups: MD-H (↑ 0.047), EX-H (↑ 0.021), MD-M (↑ 0.015). EX-M alone stable. MD-H crosses the ≥0.33 clinical threshold.
	G(Δ); A(Δ%)	MD-H vs. EX-M	—	0.066 (SE 0.010)	↑ ~10%	**<0.001**	Largest between-subgroup QUICKI differential. High adherence (cross-group): ~10% greater relative improvement.
**McAuley Index**	T×G; T×A	MD-H: T1 → T2	6.23 → 6.83	—	↑ 0.60	**0.033**	Crossover pattern: MD-H uniquely ↑; all other subgroups ↓ (EX-M steepest ↓ 1.84, *p* < 0.001; EX-H ↓ 0.91; MD-M ↓ 0.67).
	G(Δ); A(Δ%)	MD-H vs. EX-M	—	2.83 (SE 0.55)	↑ ~20%	**<0.001**	Largest between-subgroup iMcA differential. High adherence: ~20% better relative maintenance cross-group.
**β-Cell Function—SPINA-Carb Indices**							
**SPINA-Gβ (pmol/s)**	T×G; T×G×A	MD-H: T1 → T2	7.71 → 2.25	—	↓ 5.46	**<0.001**	Largest β-cell secretory capacity ↓. MD-M ↓ 3.11 (*p* < 0.001); EX-M ↓ 2.60 (*p* < 0.001). EX-H stable (*p* = 0.218): hypothesised insulin-independent disposal via exercise. T×G×A survives FDR (pFDR = 0.020).
	G(Δ%)	MD-H vs. EX-M	—	73.7%	↓	**<0.001**	MD-H exceeded EX-M (↓ 73.7%) and EX-H (↓ 60.2%) in percentage SPINA-Gβ reduction.
**SPINA-DI**	T×G; T×A	MD-H: T1 → T2	4.58 → 3.41	—	↓ 1.17	**<0.001**	Largest disposition index ↓. MD-M ↓ 0.49 (*p* < 0.001). EX-H near-stable (↓ 0.20, *p* = 0.171); EX-M stable—mechanistically distinct trajectories.
	G(Δ); A(Δ)	MD-H vs. EX-M	—	1.37 (SE 0.26)	↓	**<0.001**	Largest absolute differential. Also exceeds EX-H (↓ 1.08, *p* < 0.001). High adherence (cross-group): ↓ 0.449 (*p* = 0.008).
	G(Δ%)	MD-H vs. EX-M	—	29.9%	↓	**<0.001**	Greatest percentage disposition index normalisation.
**Lipid Profile**							
**Total Cholesterol (mg/dL)**	T×A	MD-H: T1 → T2	141 → 119	—	↓ 21.4	**<0.001**	↓ restricted to high-adherence subgroups: MD-H ↓ 21.4; EX-H ↓16.6 (167 → 150, *p* = 0.008). Medium-adherence subgroups stable.
	A(Δ); A(Δ%)	High vs. Medium (cross-group)	—	~23 mg/dL	↓ ~19%	**0.003**	High adherence: 23 mg/dL greater absolute and 19% greater relative total cholesterol reduction, cross-group.
**LDL-Cholesterol (mg/dL)**	T×G; T×A	EX-H: T1 → T2	117.1 → 68.3	—	↓ 48.8	**<0.001**	Largest absolute LDL-c reduction. MD-H ↓ 17.7 (*p* = 0.004); EX-M ↓ 18.2 (*p* = 0.002). MD-M alone no significant change, indicating a dietary adherence threshold for lipid response.
	G(Δ); A(Δ)	EX-H vs. MD-M	—	52.9 (SE 11.0)	↓	**<0.001**	Largest pairwise LDL-c differential. High adherence (cross-group): ~26 mg/dL greater absolute reduction (*p* = 0.001).
**Cardiometabolic Risk Indices**							
**TyG Index**	T	All subgroups	↑ Universal	—	MD-M ↑ 0.83; MD-H ↑ 0.74; EX-M ↑ 0.98; EX-H ↑ 0.59	**<0.001**	Universal ↑ consistent with pubertal triglyceride physiology. No group or adherence differential survived FDR.
**TG/HDL-c**	T	All subgroups	↑ Universal	—	MD-M +1.79; MD-H +3.75; EX-M +4.02; EX-H +2.60	**<0.001**	Universal ↑; T×G×A crude *p* = 0.008 did not survive FDR (pFDR = 0.050). Pubertal atherogenic floor; no differential modulation confirmed.
**TC/HDL-c**	T×G; G(Δ); G(Δ%)	EX-M: T1→T2	4.58 → 6.68	—	↑ 2.11	**<0.001**	↑ in both Exercise subgroups (MD-M ↑2.11; MD-H ↑1.51). MedDiet subgroups stable. MedDiet intervention prevented TC/HDL-c deterioration.
	G(Δ); G(Δ%)	MD-M vs. EX-M	—	4.30 (SE 1.28)	↓ 86.9%	**0.008**	MD-M showed significantly more favourable TC/HDL-c change than EX-M.
**Hepatic Biomarkers**							
**Total Bilirubin (mg/dL)**	T; A(Δ); A(Δ%)	All subgroups (universal ↓)	↓ Universal	—	↓	**<0.001**	Universal ↓ over time; values within pediatric physiological range. High adherence: greater absolute (*p* = 0.002) and percentage (*p* = 0.004) reduction cross-group.
**GGT (U/L)**	T	MD-M: T1 → T2	11.2 → 14.8	—	↑ 3.51	**<0.001**	Universal temporal ↑ (pFDR = 0.013); directionally largest in MD-M. No group × adherence differential survived FDR. In absence of ALT/AST elevation, interpreted as adaptive enzymatic response.

**Note.** Only outcomes with ≥1 FDR-adjusted significant term (Benjamini–Hochberg, pFDR < 0.05) from LMM-REML or GLM-ANCOVA are included. EMM = estimated marginal means (covariate-adjusted); T1/T2 values from Supplementary Table S4. Bonferroni-corrected *p*-values from post hoc pairwise comparisons or Simple Effects (LMM). Δ = absolute intraindividual change (T2 − T1); Δ% = [(T2 − T1)/T1 × 100]. All models adjusted for zBMI/A and zMETs. MedDiet: *n* = 46; Exercise: *n* = 32; MD-M & EX-M: KIDMED 4–7; MD-H & EX-H: KIDMED ≥ 8. T×G×A for SPINA-Gβ (pFDR = 0.020) survives FDR correction and is reported as statistically robust. Bold red values: *p* < 0.05 (Bonferroni-corrected). Adjusted Mean (T1 → T2): covariate-adjusted estimated marginal means from LMM-REML at baseline (T1) and 6-month follow-up (T2); reported only for simple effects rows. Group Difference: GLM-ANCOVA estimate of the difference in Δ or Δ% between contrasted subgroups; reported only for between-group contrast rows. Arrows: →, transition; ↑, increase; ↓, decrease.

## Data Availability

The original contributions presented in this study are included in the article/Supplementary Materials. Further inquiries can be directed to the corresponding authors.

## Supplementary Materials

The following supporting information can be downloaded at: https://www.mdpi.com/article/10.3390/nu18091453/s1, Figure S1. CONSORT participant flow diagram; Figure S2. Full LMM for glycemic control and insulin sensitivity; Figure S3. Full LMM for SPINA-Carb indices. Figure S4. Full LMM for lipid profile and cardiometabolic risk indices; Figure S5. Full LMM for hepatic biomarkers; Table S1. Spearman correlation matrix between KIDMED and metabolic parameters and prospective changes (Δ); Table S2. Linear mixed model: fixed effects for all outcomes; Table S3. General linear model (GLM-ANCOVA) on absolute intraindividual change (Δ) and normalized percentage change (Δ%): full statistics; Table S4. Estimated marginal means from the LMM. Table S5. FDR-adjusted (Benjamini–Hochberg) *p*-values for all fixed effects and interactions across outcome families.

## Abbreviations

The following abbreviations are used in this manuscript:

ALT	Alanine aminotransferase
AMPK	Adenosine monophosphate-activated protein kinase
AST	Aspartate aminotransferase
BMI	Body mass index
Δ	Absolute intraindividual change (T2 − T1)
Δ%	Normalized percentage change [(T2 − T1)/T1 × 100]
EMM	Estimated marginal means
EX	Exercise group
EX-H	High Mediterranean diet adherence Exercise subgroup
EX-M	Medium Mediterranean diet adherence Exercise subgroup
FDR	False discovery rate
GGT	Gamma-glutamyl transferase
GLM	General linear model
GLUT4	Glucose transporter type 4
HDL-c	High-density lipoprotein cholesterol
HOMA-β	Homeostasis Model Assessment of β-cell function
HOMA-IR	Homeostasis Model Assessment of Insulin Resistance
IGF-1	Insulin-like growth factor-1
IPAQ-C	International Physical Activity Questionnaire for Children
iMcA	McAuley index
IRS-1	Insulin receptor substrate-1
KIDMED	Mediterranean Diet Quality Index for children and adolescents
LDL-c	Low-density lipoprotein cholesterol
LMM	Linear mixed model
LPL	Lipoprotein lipase
MD	MedDiet group
MD-H	High adherence to Mediterranean diet
MD-M	Medium adherence to Mediterranean diet
MedDiet	Mediterranean diet
METs	Metabolic Equivalent of Task
MUFA	Monounsaturated fatty acids
Non-HDL-c	Non-high-density lipoprotein cholesterol
PA	Physical activity
QUICKI	Quantitative Insulin Sensitivity Check Index
REML	Restricted Maximum Likelihood
SEM	Standard error of the mean
SPINA	Structure-Parameter-Inference Approach
SPINA-DI	SPINA integrated disposition index
SPINA-Gβ	SPINA secretory capacity of pancreatic β-cells
SPINA-GR	SPINA insulin receptor sensitivity (receptor gain)
T1	Baseline
T2	Six-month follow-up
TC	Total cholesterol
TC/HDL-c	Total cholesterol-to-HDL cholesterol ratio
TG	Triglycerides
TG/Glu	Triglyceride–glucose index
TG/HDL-c	Triglyceride-to-HDL cholesterol ratio
VLDL	Very low-density lipoprotein
zBMI/A	Body mass index-for-age z-score (WHO reference)
zH/A	Height-for-age z-score (WHO reference)
zMETs	Standardized physical activity load (MET z-score)
