# Supplementary material for: Combined Effects of Mediterranean Diet Adherence and Physical Activity on Metabolic Homeostasis and Beta-Cell Function in Male Adolescents
Source: Nutrients. 2026 Apr 30;18(9):1453. doi: 10.3390/nu18091453 (PMC13164707; doi:10.3390/nu18091453)
Supplement: Supplementary file 1 [file nutrients-18-01453-s001.zip › Tables Supplementary.pdf]

TABLES & FIGURES SUPPLEMENTARY | NUTRIENTS-4188729

Supplementary Table S1. Spearman correlation matrices between Mediterranean diet adherence (KIDMED) and baseline metabolic parameters and prospective six-month changes (Δ = T1 − T2), stratified by intervention group.

Variable	BASELINE CORRELATIONS									PROSPECTIVE Δ CORRELATIONS								
	All			MedDiet			Exercise			All			MedDiet			Exercise		
	ρ	95% CI	p	ρ	95% CI	p	ρ	95% CI	p	ρ	95% CI	p	ρ	95% CI	p	ρ	95% CI	p
**Glycemic Control & Insulin Sensitivity**																		
Glucose (mg/dL)	.187	−.052; .406	.113	.291	−.023; .553	.061	.091	−.282; .441	.625	.030	−.321; .374	.866	−.004	−.469; .463	.988	.098	−.450; .592	.728
Insulin (µUI/mL)	−.449	−.638; −.208	**<.001**	−.483	−.723; −.137	**.007**	−.523	−.755; −.175	**.004**	−.069	−.434; .315	.721	−.581	−.847; −.081	**.023**	.130	−.444; .629	.657
HOMA-IR	−.393	−.598; −.139	**.003**	−.411	−.682; −.041	**.027**	−.456	−.714; −.089	**.015**	−.111	−.474; .284	.573	−.576	−.853; −.048	**.031**	.047	−.509; .575	.873
HOMA-β (%)	−.479	−.662; −.242	**<.001**	−.465	−.716; −.107	**.011**	−.522	−.754; −.174	**.004**	.054	−.336; .428	.784	−.239	−.692; .350	.411	.278	−.312; .714	.335
QUICKI	.389	.135; .595	**.003**	.421	.053; .688	**.023**	.437	.065; .702	**.020**	.275	−.121; .595	.157	.559	.024; .846	**.038**	.136	−.439; .632	.642
McAuley Index (iMcA)	.380	.125; .588	**.004**	.339	−.042; .635	.072	.411	.033; .686	**.030**	.277	−.118; .597	.153	.509	−.047; .824	.063	.193	−.391; .666	.508
**β-cell Function & Insulin Sensitivity (SPINA-Carb Indices)**																		
SPINA-Gβ (pmol/s)	−.477	−.660; −.239	**<.001**	−.490	−.731; −.139	**.007**	−.541	−.765; −.199	**.003**	.023	−.363; .403	.907	−.417	−.783; .163	.138	.285	−.305; .717	.323
SPINA-GR (mol/s)	−.415	−.614; −.165	**.001**	−.474	−.721; −.118	**.009**	−.334	−.636; .056	.082	.111	−.284; .474	.573	.048	−.508; .576	.870	.171	−.410; .653	.560
SPINA-DI	−.478	−.661; −.241	**<.001**	−.465	−.716; −.107	**.011**	−.522	−.754; −.174	**.004**	−.062	−.435; .329	.754	−.509	−.824; .047	.063	.352	−.236; .752	.216
**Lipid Profile**																		
Triglycerides (mg/dL)	−.094	−.323; .146	.431	−.035	−.344; .280	.823	−.161	−.496; .216	.194	−.218	−.525; .140	.216	.037	−.436; .494	.881	−.560	−.838; −.050	**.030**
Total Cholesterol (mg/dL)	−.087	−.317; .152	.463	−.105	−.404; .215	.509	−.149	−.486; .228	.212	−.262	−.559; .094	.134	−.249	−.640; .245	.305	−.256	−.688; .310	.357
LDL-Cholesterol (mg/dL)	.273	.045; .473	**.020**	.467	.190; .675	**<.001**	−.009	−.293; .420	.482	−.425	−.640; −.033	**.014**	−.330	−.696; .148	.168	−.511	−.811; .002	.052
HDL-Cholesterol (mg/dL)	−.313	−.507; −.090	**.007**	−.388	−.619; −.095	**.006**	−.188	−.492; .208	.156	−.434	−.669; −.083	**.010**	−.679	−.866; −.325	**.001**	−.087	−.574; .445	.757
Non-HDL-Cholesterol (mg/dL)	.017	−.214; .246	.884	.032	−.274; .333	.838	−.079	−.214; .487	.675	−.014	−.326; .351	.937	.094	−.376; .526	.702	−.142	−.610; .400	.614
**Cardiometabolic Risk Indices**																		
TG/HDL-c	.114	−.126; .342	.336	.200	−.120; .482	.204	−.029	−.389; .339	.878	.135	−.223; .461	.447	.349	−.139; .701	.143	−.287	−.705; .280	.300
TyG Index	−.018	−.254; .219	.879	.032	−.283; .341	.840	−.058	−.413; .312	.755	−.151	−.473; .208	.395	.010	−.458; .474	.967	−.245	−.682; .320	.378
TC/HDL-c	.250	.014; .459	**.033**	.277	−.039; .542	.076	.116	−.259; .460	.535	.361	.016; .630	**.036**	.494	.036; .780	**.032**	−.080	−.580; .464	.777
**Hepatic Biomarkers**																		
Total Bilirubin (mg/dL)	.080	−.160; .311	.501	.106	−.214; .405	.505	.014	−.351; .376	.939	−.594	−.781; −.311	**<.001**	−.496	−.781; −.040	**.031**	−.673	−.885; −.230	**.006**
AST (U/L)	−.138	−.363; .101	.243	−.253	−.283; .341	.105	−.023	−.384; .344	.902	.082	−.273; .418	.644	.156	−.334; .579	.525	.136	−.418; .617	.628
ALT (U/L)	.007	−.230; .244	.951	.126	−.194; .422	.425	−.189	−.518; .188	.308	−.260	−.557; .096	.138	−.334	−.692; .155	.162	−.158	−.630; .400	.574
GGT (U/L)	.035	−.204; .269	.770	.165	−.155; .454	.296	−.208	−.532; .169	.261	−.184	−.499; .174	.297	−.043	−.499; .431	.860	−.131	−.613; .423	.642

Abbreviations: ρ (rho), Spearman rank correlation coefficient; 95% CI, 95% confidence interval; Δ, absolute intraindividual change (T1 − T2); MedDiet, Mediterranean Diet group; Exercise, Exercise group; iMcA, McAuley index; TG/HDL-c, triglycerides/HDL cholesterol ratio; TyG, triglyceride–glucose index; TC/HDL-c, total cholesterol/HDL ratio; SPINA-Gβ, β-cell secretory capacity; SPINA-GR, insulin receptor sensitivity; SPINA-DI, disposition index; AST, aspartate aminotransferase; ALT, alanine aminotransferase; GGT, gamma-glutamyl transferase.

Interpretation: Values in bold red indicate $p < .05$. Baseline correlations (S1) reflect associations between KIDMED score and metabolic parameters measured at T1. Prospective Δ correlations (S2) reflect associations between baseline KIDMED score and six-month intraindividual changes in each outcome. All analyses performed using Spearman's rank correlation.

Supplementary Table S2. Linear mixed model (REML): fixed effects for all outcomes.

F-statistics and p-values for all nine fixed effects per outcome variable, with marginal and conditional R^2 reported by variable. Model includes random intercepts for individual ID and covariates zBMI/A and zMETs.

Variable	Source of Variation	F (1, df)	p-value	R^2marginal	R^2conditional
**Glycemic Control & Insulin Sensitivity**					
Glucose	Time	0.42 (106)	.519	.040	.392
	Group	0.12 (32)	.735		
	Adherence	2.08 (32)	.159		
	zBMI/A	0.01 (35)	.941		
	zMETs	0.09 (32)	.761		
	Time × Group	0.13 (106)	.724		
	Time × Adherence	0.83 (106)	.363		
	Group × Adherence	0.34 (33)	.563		
	Time × G × A†	0.08 (106)	.783		
Insulin	**Time**	**36.06 (83)**	**<.001**	.307	.912
	Group	0.01 (23)	.913		
	**Adherence**	**7.63 (23)**	**.011**		
	zBMI/A	0.09 (23)	.768		
	zMETs	4.05 (23)	.056		
	**Time × Group**	**11.03 (83)**	**.001**		
	**Time × Adherence**	**5.28 (83)**	**.024**		
	Group × Adherence	0.40 (23)	.531		
	Time × G × A†	**5.44 (83)**	**.022**		
HOMA-IR	**Time**	**11.31 (82)**	**.001**	.333	.750
	Group	0.09 (23)	.771		
	**Adherence**	**9.78 (23)**	**.005**		
	zBMI/A	<0.01 (23)	.994		
	**zMETs**	**4.95 (23)**	**.036**		
	**Time × Group**	**6.10 (82)**	**.016**		
	**Time × Adherence**	**4.72 (82)**	**.033**		
	Group × Adherence	0.29 (23)	.593		
	Time × G × A†	1.88 (82)	.174		
HOMA-β	**Time**	**37.25 (81)**	**<.001**	.238	.909
	Group	0.12 (23)	.737		
	**Adherence**	**5.22 (23)**	**.032**		
	zBMI/A	0.52 (23)	.479		
	zMETs	2.74 (23)	.111		
	Time × Group	3.41 (81)	.068		
	Time × Adherence	0.16 (81)	.686		
	Group × Adherence	0.29 (23)	.597		
	Time × G × A†	3.63 (81)	.060		
QUICKI	**Time**	**63.53 (82)**	**<.001**	.475	.857
	Group	1.41 (23)	.248		
	**Adherence**	**13.31 (23)**	**.001**		
	zBMI/A	0.04 (23)	.836		

Variable	Source of Variation	F (1, df)	p-value	R^2marginal	R^2conditional
	zMETs	4.19 (23)	.052		
	**Time × Group**	**14.00 (82)**	**<.001**		
	**Time × Adherence**	**23.33 (82)**	**<.001**		
	Group × Adherence	1.41 (23)	.246		
	Time × G × A†	1.28 (82)	.261		
iMcA	**Time**	**25.97 (81)**	**<.001**	.422	.867
	Group	0.97 (23)	.334		
	**Adherence**	**9.92 (23)**	**.004**		
	zBMI/A	<0.01 (23)	.950		
	zMETs	4.06 (23)	.056		
	**Time × Group**	**23.39 (81)**	**<.001**		
	**Time × Adherence**	**15.67 (81)**	**<.001**		
	Group × Adherence	2.26 (23)	.146		
	Time × G × A†	0.37 (81)	.543		
**β-cell Function & Insulin Sensitivity — SPINA-Carb Indices**					
SPINA-Gβ	**Time**	**56.73 (81)**	**<.001**	.270	.938
	Group	0.06 (23)	.815		
	**Adherence**	**6.16 (23)**	**.021**		
	zBMI/A	0.37 (23)	.547		
	zMETs	3.19 (23)	.087		
	**Time × Group**	**9.02 (81)**	**.004**		
	Time × Adherence	0.26 (81)	.610		
	Group × Adherence	0.30 (23)	.590		
	Time × G × A†	**5.63 (81)**	**.020**		
SPINA-GR	**Time**	**5.93 (82)**	**.017**	.172	.760
	Group	0.06 (23)	.805		
	**Adherence**	**4.64 (23)**	**.042**		
	zBMI/A	0.53 (23)	.474		
	zMETs	1.45 (23)	.240		
	Time × Group	2.77 (82)	.100		
	Time × Adherence	1.31 (82)	.255		
	Group × Adherence	0.13 (23)	.721		
	Time × G × A†	1.27 (82)	.263		
SPINA-DI	**Time**	**54.23 (81)**	**<.001**	.427	.895
	Group	0.58 (23)	.453		
	**Adherence**	**12.68 (23)**	**.002**		
	zBMI/A	0.14 (23)	.713		
	zMETs	3.68 (23)	.067		
	**Time × Group**	**26.15 (81)**	**<.001**		
	**Time × Adherence**	**8.50 (81)**	**.005**		
	Group × Adherence	0.71 (23)	.407		
	Time × G × A†	**4.82 (81)**	**.031**		
**Lipid Profile**					
Triglycerides	**Time**	**91.31 (102)**	**<.001**	.367	.563
	Group	0.39 (28)	.539		

Variable	Source of Variation	F (1, df)	p-value	R^2marginal	R^2conditional
	Adherence	2.31 (28)	.140		
	zBMI/A	0.19 (31)	.664		
	zMETs	0.05 (28)	.828		
	Time × Group	0.16 (102)	.693		
	Time × Adherence	2.83 (102)	.096		
	Group × Adherence	0.21 (29)	.651		
	Time × G × A†	**4.77 (102)**	**.031**		
Total Cholesterol	**Time**	**9.95 (102)**	**.002**	.232	.773
	**Group**	**5.91 (31)**	**.021**		
	Adherence	1.26 (31)	.271		
	**zBMI/A**	**8.07 (33)**	**.008**		
	zMETs	0.33 (31)	.573		
	Time × Group	1.26 (102)	.265		
	**Time × Adherence**	**12.42 (102)**	**<.001**		
	Group × Adherence	0.03 (32)	.863		
	Time × G × A†	0.08 (102)	.783		
LDL-c	**Time**	**51.82 (105)**	**<.001**	.322	.591
	Group	2.98 (32)	.094		
	Adherence	0.81 (32)	.374		
	zBMI/A	0.39 (35)	.536		
	zMETs	0.09 (32)	.764		
	**Time × Group**	**22.15 (105)**	**<.001**		
	**Time × Adherence**	**21.38 (105)**	**<.001**		
	Group × Adherence	1.23 (33)	.276		
	Time × G × A†	0.68 (105)	.412		
HDL-c	Time	0.82 (105)	.369	.201	.541
	Group	1.55 (32)	.222		
	**Adherence**	**7.62 (32)**	**.010**		
	zBMI/A	2.71 (34)	.109		
	zMETs	3.06 (32)	.090		
	Time × Group	1.72 (105)	.193		
	Time × Adherence	3.66 (105)	.059		
	Group × Adherence	0.38 (33)	.541		
	Time × G × A†	2.24 (105)	.137		
Non-HDL-c	**Time**	**6.02 (102)**	**.016**	.184	.707
	**Group**	**4.49 (31)**	**.042**		
	Adherence	0.05 (31)	.819		
	**zBMI/A**	**5.90 (32)**	**.021**		
	zMETs	<0.01 (31)	.986		
	Time × Group	2.79 (102)	.098		
	**Time × Adherence**	**5.37 (102)**	**.022**		
	Group × Adherence	<0.01 (31)	.969		
	Time × G × A†	1.07 (102)	.303		
**Cardiometabolic Risk Indices**					
TyG Index	**Time**	**130.30 (102)**	**<.001**	.395	.642

Variable	Source of Variation	F (1, df)	p-value	R^2marginal	R^2conditional
	Group	0.36 (29)	.555		
	Adherence	0.41 (28)	.529		
	zBMI/A	0.13 (31)	.722		
	zMETs	0.09 (29)	.771		
	Time × Group	<0.01 (102)	.967		
	Time × Adherence	3.05 (102)	.084		
	Group × Adherence	0.17 (30)	.687		
	Time × G × A†	1.16 (102)	.283		
	**Time**	**95.02 (105)**	**<.001**		
	Group	0.01 (31)	.929		
	Adherence	0.44 (31)	.510		
	zBMI/A	0.04 (34)	.844		
TG/HDL-c	zMETs	0.03 (31)	.854	.344	.543
	Time × Group	0.76 (105)	.385		
	Time × Adherence	0.19 (105)	.666		
	Group × Adherence	0.56 (32)	.459		
	Time × G × A†	**7.38 (105)**	**.008**		
	**Time**	**7.03 (104)**	**.009**		
	Group	1.68 (32)	.204		
	Adherence	1.64 (32)	.209		
	zBMI/A	0.01 (34)	.907		
TC/HDL-c	zMETs	0.38 (32)	.543	.168	.537
	**Time × Group**	**12.38 (104)**	**<.001**		
	Time × Adherence	0.22 (104)	.637		
	Group × Adherence	0.10 (33)	.751		
	Time × G × A†	2.21 (104)	.140		
**Hepatic Biomarkers**					
	**Time**	**19.21 (109)**	**<.001**		
	Group	0.83 (31)	.369		
	Adherence	0.67 (31)	.419		
	zBMI/A	0.10 (36)	.749		
Total Bilirubin	zMETs	0.002 (31)	.967	.401	.625
	Time × Group	0.31 (109)	.577		
	Time × Adherence	0.70 (109)	.406		
	Group × Adherence	0.05 (33)	.832		
	Time × G × A†	0.04 (109)	.844		
	Time	1.76 (106)	.187		
	Group	0.73 (33)	.399		
	Adherence	3.35 (32)	.076		
	zBMI/A	0.42 (35)	.521		
AST	zMETs	0.67 (33)	.420	.138	.429
	Time × Group	2.20 (106)	.141		
	Time × Adherence	2.66 (106)	.106		
	Group × Adherence	0.02 (34)	.883		
	Time × G × A†	**4.58 (106)**	**.035**		

Variable	Source of Variation	F (1, df)	p-value	R^2marginal	R^2conditional
ALT	Time	0.43 (99)	.514	.109	.675
	Group	0.01 (28)	.911		
	Adherence	0.41 (28)	.529		
	zBMI/A	0.41 (29)	.528		
	zMETs	0.77 (28)	.389		
	Time × Group	0.44 (99)	.506		
	Time × Adherence	2.31 (99)	.132		
	Group × Adherence	2.10 (29)	.158		
	Time × G × A†	**4.87 (99)**	**.030**		
GGT	**Time**	**11.06 (103)**	**.001**	.126	.630
	Group	0.01 (32)	.940		
	Adherence	0.18 (31)	.673		
	zBMI/A	0.15 (33)	.703		
	zMETs	0.02 (32)	.884		
	Time × Group	1.05 (103)	.309		
	Time × Adherence	1.84 (103)	.178		
	**Group × Adherence**	**4.42 (32)**	**.043**		
	Time × G × A†	1.92 (103)	.168		

Note. Linear mixed model (REML) with random intercepts for individual ID. F(1, df) = F-statistic with 1 numerator degree of freedom; denominator df via Satterthwaite approximation. R^2m = marginal R^2; R^2c = conditional R^2 (both reported once per variable). Covariates: zBMI/A (BMI-for-age z-score) and zMETs (standardized physical activity). † Time × Group × Adherence (three-way interaction). Values in bold red: $p < .05$.

Supplementary Table S3. General linear model (ANCOVA): Group × Adherence effects on absolute (Δ) and percentage (Δ%) change — side-by-side comparison.

Results from two parallel ANCOVA models (Type III SS) for each outcome: one on absolute change (Δ, teal columns) and one on normalized percentage change (Δ%, purple columns), adjusting for zBMI/A and zMETs. Adj.R^2 reported per variable.

Variable	Source of Variation	GLM on Absolute Δ				GLM on Percentage Δ%				Adj.R^2	
		F(df)	p	β	η^2p	F(df)	p	β	η^2p	Δ	Δ%
**Glycemic Control & Insulin Sensitivity**											
Glucose	Group	0.22 (1,60)	.642	0.15	.004	0.47 (1,60)	.496	0.22	.008	.000	.000
	Adherence	0.87 (1,60)	.355	−0.24	.014	0.42 (1,60)	.519	−0.17	.007		
	zBMI/A	<0.01 (1,60)	.977	0.00	<.001	0.56 (1,60)	.458	−0.11	.009		
	zMETs	1.84 (1,60)	.180	−0.21	.030	1.84 (1,60)	.180	−0.21	.030		
	Group × Adherence††	0.05 (1,60)	.826	−0.12	<.001	<0.01 (1,60)	.955	−0.03	<.001		
Insulin	**Group**	**7.45 (1,52)**	**.009**	**0.82**	**.125**	**18.29 (1,52)**	**<.001**	**1.14**	**.260**	.148	.330
	**Adherence**	3.62 (1,52)	.063	−0.48	.065	**14.07 (1,52)**	**<.001**	**−0.83**	**.213**		
	zBMI/A	0.07 (1,52)	.792	−0.04	<.001	0.09 (1,52)	.769	0.04	.002		
	zMETs	1.18 (1,52)	.283	−0.17	.022	4.01 (1,52)	.050	−0.27	.072		
	Group × Adherence††	2.96 (1,52)	.091	0.87	.054	1.26 (1,52)	.268	0.50	.024		
HOMA-IR	**Group**	**5.27 (1,50)**	**.026**	**0.74**	**.095**	**16.95 (1,50)**	**<.001**	**1.12**	**.253**	.090	.337
	**Adherence**	3.44 (1,50)	.070	−0.48	.064	**17.38 (1,50)**	**<.001**	**−0.92**	**.258**		
	zBMI/A	0.11 (1,50)	.739	−0.05	.002	0.70 (1,50)	.406	0.10	.014		
	**zMETs**	1.98 (1,50)	.166	−0.23	.038	**7.33 (1,50)**	**.009**	**−0.37**	**.128**		
	Group × Adherence††	0.91 (1,50)	.346	0.52	.018	0.08 (1,50)	.776	−0.13	.002		
HOMA-β	**Group**	1.57 (1,50)	.216	0.41	.031	**13.76 (1,50)**	**<.001**	**1.10**	**.216**	.043	.221
	Adherence	0.03 (1,50)	.854	0.05	<.001	1.49 (1,50)	.228	−0.29	.029		
	zBMI/A	2.95 (1,50)	.092	0.25	.056	0.02 (1,50)	.898	0.02	<.001		
	zMETs	0.39 (1,50)	.538	0.10	.008	0.60 (1,50)	.443	−0.12	.012		
	Group × Adherence††	1.07 (1,50)	.307	0.57	.021	2.01 (1,50)	.163	0.71	.039		
QUICKI	**Group**	**24.28 (1,50)**	**<.001**	**−1.25**	**.327**	**23.27 (1,50)**	**<.001**	**−1.24**	**.318**	.428	.410
	**Adherence**	**23.23 (1,50)**	**<.001**	**0.99**	**.317**	**20.53 (1,50)**	**<.001**	**0.94**	**.291**		
	zBMI/A	3.10 (1,50)	.084	−0.20	.058	2.67 (1,50)	.109	−0.19	.051		
	**zMETs**	**10.04 (1,50)**	**.003**	**0.41**	**.167**	**8.74 (1,50)**	**.005**	**0.39**	**.149**		
	Group × Adherence††	<0.01 (1,50)	.959	0.02	<.001	0.11 (1,50)	.743	−0.14	.002		
iMcA	**Group**	**15.21 (1,50)**	**<.001**	**−1.06**	**.233**	**9.39 (1,50)**	**.004**	**−0.87**	**.158**	.344	.283
	**Adherence**	**12.42 (1,50)**	**<.001**	**0.78**	**.199**	**15.66 (1,50)**	**<.001**	**0.91**	**.239**		
	**zBMI/A**	**5.27 (1,50)**	**.026**	**−0.28**	**.095**	1.98 (1,50)	.166	−0.18	.038		
	zMETs	0.05 (1,50)	.817	0.03	<.001	0.43 (1,50)	.517	0.09	.008		
	Group × Adherence††	0.07 (1,50)	.793	0.12	<.001	0.19 (1,50)	.662	0.21	.004		
**β-cell Function & Insulin Sensitivity — SPINA-Carb Indices**											
SPINA-Gβ	**Group**	**5.18 (1,50)**	**.027**	**0.73**	**.094**	**18.74 (1,50)**	**<.001**	**1.22**	**.273**	.100	.289
	**Adherence**	0.34 (1,50)	.563	−0.15	.007	**5.94 (1,50)**	**.018**	**−0.56**	**.106**		
	zBMI/A	2.03 (1,50)	.161	0.20	.039	0.35 (1,50)	.559	0.07	.007		
	zMETs	<0.01 (1,50)	.989	<−0.01	<.001	2.24 (1,50)	.141	−0.22	.043		
	Group × Adherence††	1.88 (1,50)	.177	0.74	.036	0.95 (1,50)	.336	0.46	.019		

Variable	Source of Variation	GLM on Absolute Δ				GLM on Percentage Δ%				Adj.R^2	
		F(df)	p	β	η^2p	F(df)	p	β	η^2p	Δ	Δ%
SPINA-GR	Group	0.43 (1,50)	.516	0.22	.008	1.35 (1,50)	.251	0.38	.026	.019	.035
	Adherence	0.82 (1,50)	.369	0.24	.016	0.74 (1,50)	.394	0.23	.015		
	zBMI/A	1.64 (1,50)	.207	0.19	.032	0.35 (1,50)	.558	0.09	.007		
	zMETs	1.62 (1,50)	.209	0.22	.031	1.28 (1,50)	.263	0.19	.025		
	Group × Adherence††	0.43 (1,50)	.513	0.37	.009	0.88 (1,50)	.353	0.52	.017		
SPINA-DI	**Group**	**20.88 (1,50)**	**<.001**	**1.24**	**.295**	**20.36 (1,50)**	**<.001**	**1.24**	**.289**	.338	.323
	**Adherence**	**7.60 (1,50)**	**.008**	**−0.61**	**.132**	**5.97 (1,50)**	**.018**	**−0.55**	**.107**		
	zBMI/A	3.76 (1,50)	.058	0.24	.070	2.20 (1,50)	.145	0.18	.042		
	zMETs	1.48 (1,50)	.230	−0.17	.029	1.32 (1,50)	.257	−0.16	.026		
	Group × Adherence††	0.91 (1,50)	.345	0.44	.018	1.41 (1,50)	.241	0.55	.027		
**Lipid Profile**											
Triglycerides	Group	0.33 (1,60)	.570	−0.18	.005	1.39 (1,60)	.242	−0.36	.023	.013	.059
	Adherence	2.20 (1,60)	.143	−0.38	.035	2.62 (1,60)	.111	−0.40	.042		
	zBMI/A	0.55 (1,60)	.461	0.11	.009	1.37 (1,60)	.247	0.16	.022		
	zMETs	0.68 (1,60)	.413	0.13	.011	3.95 (1,60)	.051	0.30	.062		
	Group × Adherence††	2.60 (1,60)	.112	−0.87	.042	0.04 (1,60)	.838	−0.11	<.001		
Total Cholesterol	**Group**	**4.32 (1,60)**	**.042**	**0.62**	**.067**	**6.28 (1,60)**	**.015**	**0.73**	**.095**	.112	.147
	**Adherence**	**9.83 (1,60)**	**.003**	**−0.75**	**.141**	**10.66 (1,60)**	**.002**	**−0.77**	**.151**		
	zBMI/A	0.07 (1,60)	.797	0.04	<.001	0.05 (1,60)	.820	−0.03	<.001		
	**zMETs**	**4.58 (1,60)**	**.036**	**−0.31**	**.071**	**4.78 (1,60)**	**.033**	**−0.31**	**.074**		
	Group × Adherence††	0.26 (1,60)	.614	−0.26	.004	0.53 (1,60)	.470	−0.36	.009		
LDL-c	**Group**	**8.54 (1,60)**	**.005**	**−0.80**	**.125**	**5.26 (1,60)**	**.025**	**−0.68**	**.081**	.255	.121
	**Adherence**	**11.70 (1,60)**	**.001**	**−0.75**	**.163**	**4.61 (1,60)**	**.036**	**−0.51**	**.071**		
	zBMI/A	0.01 (1,60)	.907	−0.01	<.001	1.13 (1,60)	.291	−0.14	.019		
	zMETs	<0.01 (1,60)	.983	<−0.01	<.001	0.02 (1,60)	.893	0.02	<.001		
	Group × Adherence††	0.37 (1,60)	.544	−0.29	.006	1.00 (1,60)	.322	−0.51	.016		
HDL-c	Group	1.51 (1,60)	.224	−0.38	.025	1.44 (1,60)	.235	−0.34	.023	.052	.182
	**Adherence**	2.17 (1,60)	.146	−0.36	.035	**6.54 (1,60)**	**.013**	**−0.59**	**.098**		
	zBMI/A	1.99 (1,60)	.164	−0.20	.032	0.36 (1,60)	.549	0.08	.006		
	zMETs	0.01 (1,60)	.938	−0.01	<.001	0.01 (1,60)	.911	−0.02	<.001		
	Group × Adherence††	3.03 (1,60)	.087	0.92	.048	**4.27 (1,60)**	**.043**	**1.01**	**.066**		
Non-HDL-c	**Group**	**6.53 (1,60)**	**.013**	**0.78**	**.098**	**6.03 (1,60)**	**.017**	**0.73**	**.091**	.084	.118
	**Adherence**	**4.73 (1,60)**	**.034**	**−0.53**	**.073**	**4.92 (1,60)**	**.030**	**−0.53**	**.076**		
	zBMI/A	0.91 (1,60)	.345	0.13	.015	0.45 (1,60)	.504	−0.09	.007		
	zMETs	3.81 (1,60)	.056	−0.29	.060	3.59 (1,60)	.063	−0.28	.056		
	Group × Adherence††	1.82 (1,60)	.182	−0.70	.029	1.50 (1,60)	.225	−0.62	.024		
**Cardiometabolic Risk Indices**											
TG/HDL-c	Group	0.35 (1,60)	.555	0.19	.006	0.36 (1,60)	.550	0.19	.006	.022	.000
	Adherence	0.04 (1,60)	.847	0.05	<.001	<0.01 (1,60)	.997	<0.01	<.001		
	zBMI/A	0.92 (1,60)	.341	0.14	.015	3.13 (1,60)	.082	0.25	.050		
	zMETs	0.16 (1,60)	.694	0.06	.003	1.16 (1,60)	.285	0.17	.019		
	Group × Adherence††	**5.01 (1,60)**	**.029**	**−1.20**	**.077**	0.57 (1,60)	.453	−0.41	.009		

Variable	Source of Variation	GLM on Absolute Δ				GLM on Percentage Δ%				Adj.R^2	
		F(df)	p	β	η^2p	F(df)	p	β	η^2p	Δ	Δ%
TyG Index	Group	0.18 (1,60)	.670	−0.14	.003	0.25 (1,60)	.617	−0.16	.004	.000	.000
	Adherence	2.15 (1,60)	.148	−0.37	.035	2.20 (1,60)	.143	−0.38	.035		
	zBMI/A	0.41 (1,60)	.525	0.09	.007	0.49 (1,60)	.488	0.10	.008		
	zMETs	1.05 (1,60)	.310	0.16	.017	1.35 (1,60)	.251	0.18	.022		
	Group × Adherence††	0.63 (1,60)	.430	−0.43	.010	0.28 (1,60)	.598	−0.29	.005		
TC/HDL-c	**Group**	**10.28 (1,60)**	**.002**	**0.95**	**.146**	**10.92 (1,60)**	**.002**	**0.98**	**.154**	.121	.131
	Adherence	0.03 (1,60)	.865	0.04	<.001	<0.01 (1,60)	1.000	<0.01	<.001		
	zBMI/A	0.32 (1,60)	.573	0.08	.005	0.28 (1,60)	.597	0.07	.005		
	zMETs	1.85 (1,60)	.179	−0.20	.030	1.77 (1,60)	.189	−0.19	.029		
	Group × Adherence††	2.30 (1,60)	.134	−0.77	.037	2.37 (1,60)	.129	−0.78	.038		
**Hepatic Biomarkers**											
Total Bilirubin	Group	4.23 (1,60)	.044	0.56	.066	1.21 (1,60)	.276	0.31	.020	.270	.215
	**Adherence**	**18.80 (1,60)**	**<.001**	**−0.94**	**.239**	**15.75 (1,60)**	**<.001**	**−0.89**	**.208**		
	zBMI/A	0.97 (1,60)	.330	0.12	.016	1.09 (1,60)	.301	0.13	.018		
	**zMETs**	**7.57 (1,60)**	**.008**	**−0.36**	**.112**	3.33 (1,60)	.073	−0.25	.053		
	Group × Adherence††	1.47 (1,60)	.231	0.56	.024	1.17 (1,60)	.284	0.52	.019		
AST	Group	0.47 (1,60)	.494	0.21	.008	0.85 (1,60)	.361	0.29	.014	.116	.039
	Adherence	0.91 (1,60)	.345	0.23	.015	0.17 (1,60)	.683	0.10	.003		
	zBMI/A	2.83 (1,60)	.098	0.23	.045	1.89 (1,60)	.174	0.19	.031		
	**zMETs**	**4.22 (1,60)**	**.044**	**−0.30**	**.066**	**4.05 (1,60)**	**.049**	**−0.31**	**.063**		
	Group × Adherence††	0.48 (1,60)	.491	0.35	.008	0.08 (1,60)	.782	−0.15	<.001		
ALT	Group	0.02 (1,60)	.877	0.05	<.001	0.06 (1,60)	.805	−0.08	<.001	.030	.013
	Adherence	1.11 (1,60)	.296	−0.26	.018	0.06 (1,60)	.813	−0.06	<.001		
	zBMI/A	2.86 (1,60)	.096	−0.24	.045	2.92 (1,60)	.093	−0.24	.046		
	zMETs	0.34 (1,60)	.562	−0.09	.006	0.02 (1,60)	.880	0.02	<.001		
	Group × Adherence††	**4.11 (1,60)**	**.047**	**1.08**	**.064**	**4.64 (1,60)**	**.035**	**1.16**	**.072**		
GGT	Group	1.21 (1,60)	.277	−0.35	.020	4.01 (1,60)	.050	−0.64	.063	.008	.000
	Adherence	0.78 (1,60)	.380	−0.22	.013	0.04 (1,60)	.849	−0.05	<.001		
	zBMI/A	2.10 (1,60)	.153	−0.21	.034	0.80 (1,60)	.374	−0.13	.013		
	zMETs	0.01 (1,60)	.919	−0.02	<.001	0.23 (1,60)	.633	0.07	.004		
	Group × Adherence††	2.01 (1,60)	.162	0.76	.032	0.03 (1,60)	.873	0.09	<.001		

Note. General linear model (ANCOVA; Type III SS). Fixed factors: Group and Adherence. Continuous covariates: zBMI/A and zMETs (centered). Δ = T2 − T1 (absolute intraindividual change); Δ% = [(T1 − T2)/T1] × 100 (normalized percentage change). F(df) = F-statistic with degrees of freedom (numerator, denominator). β = standardized regression coefficient. η^2p = partial eta-squared. †† Group × Adherence interaction. Adj.R^2 = adjusted R^2 for the full model, reported once per variable. Values in bold red: $p < .05$.

Supplementary Table S4. Estimated marginal means (baseline and 6-month follow-up) from the linear mixed model.

Covariate-adjusted means for each Group × Adherence subgroup at T1 (purple) and T2 (teal), with standard errors and 95% confidence intervals. Values are model-derived estimates adjusted for zBMI/A and zMETs at their grand mean.

Variable	Group	Adherence	T1 — Baseline				T2 — 6-Month Follow-up			
			Mean	SE	95%CI Low	95%CI High	Mean	SE	95%CI Low	95%CI High
**Glycemic Control & Insulin Sensitivity**										
Glucose (mg/dL)	MedDiet	Medium	94.9	4.60	85.7	104.1	95.8	4.60	86.6	105.0
	MedDiet	High	100.5	5.38	89.7	111.3	98.1	5.83	86.5	109.7
	Exercise	Medium	90.4	5.93	78.5	102.3	90.9	6.09	78.7	103.1
	Exercise	High	102.9	5.86	91.1	114.7	97.3	5.86	85.5	109.1
Insulin (μUI/mL)	MedDiet	Medium	38.89	7.99	22.42	55.36	32.08	7.99	15.61	48.55
	MedDiet	High	28.86	8.42	11.51	46.21	8.63	8.42	−8.73	25.98
	Exercise	Medium	43.62	8.61	25.85	61.39	39.68	8.61	21.91	57.45
	Exercise	High	16.61	8.82	−1.58	34.80	12.77	8.82	−5.42	30.96
HOMA-IR	MedDiet	Medium	9.25	1.65	5.89	12.61	8.00	1.60	4.73	11.27
	MedDiet	High	7.26	1.69	3.81	10.71	2.14	1.69	−1.32	5.59
	Exercise	Medium	8.95	1.72	5.43	12.47	8.90	1.72	5.37	12.42
	Exercise	High	3.77	1.77	0.15	7.40	2.85	1.77	−0.77	6.47
HOMA-β (%)	MedDiet	Medium	164.5	44.4	73.2	255.8	113.8	43.9	23.1	204.5
	MedDiet	High	100.5	46.4	4.9	196.1	26.2	46.4	−69.4	121.8
	Exercise	Medium	206.6	47.5	108.6	304.6	155.0	47.5	57.0	253.0
	Exercise	High	65.1	48.5	−35.0	165.2	49.8	48.5	−50.3	149.9
QUICKI	MedDiet	Medium	0.285	0.010	0.265	0.304	0.300	0.009	0.280	0.319
	MedDiet	High	0.291	0.010	0.270	0.312	0.338	0.010	0.318	0.359
	Exercise	Medium	0.294	0.010	0.273	0.315	0.295	0.010	0.274	0.316
	Exercise	High	0.328	0.010	0.307	0.350	0.350	0.010	0.328	0.371
McAuley Index (iMcA)	MedDiet	Medium	6.01	0.55	4.88	7.14	5.34	0.54	4.23	6.45
	MedDiet	High	6.23	0.57	5.06	7.41	6.83	0.57	5.66	8.00
	Exercise	Medium	6.42	0.58	5.22	7.62	4.58	0.58	3.38	5.78
	Exercise	High	8.43	0.60	7.20	9.66	7.51	0.60	6.29	8.74

Variable	Group	Adherence	T1 — Baseline				T2 — 6-Month Follow-up			
			Mean	SE	95%CI Low	95%CI High	Mean	SE	95%CI Low	95%CI High
**β-cell Function & Insulin Sensitivity (SPINA-Carb Indices)**										
SPINA-Gβ (pmol/s)	MedDiet	Medium	11.76	2.74	6.11	17.41	8.65	2.72	3.03	14.27
	MedDiet	High	7.71	2.87	1.78	13.64	2.25	2.87	−3.68	8.18
	Exercise	Medium	13.79	2.95	7.71	19.87	11.19	2.95	5.11	17.27
	Exercise	High	4.79	3.01	−1.41	11.00	3.71	3.01	−2.50	9.91
SPINA-GR (mol/s)	MedDiet	Medium	14.3	0.86	12.49	16.01	13.2	0.84	11.51	14.99
	MedDiet	High	12.8	0.89	10.97	14.63	11.8	0.89	9.96	13.64
	Exercise	Medium	14.7	0.91	12.80	16.54	13.9	0.91	12.05	15.84
	Exercise	High	12.1	0.93	10.14	14.04	12.4	0.93	10.51	14.34
SPINA-DI	MedDiet	Medium	5.03	0.30	4.41	5.64	4.53	0.30	3.92	5.14
	MedDiet	High	4.58	0.31	3.93	5.22	3.41	0.31	2.76	4.05
	Exercise	Medium	4.81	0.32	4.15	5.47	4.71	0.32	4.05	5.37
	Exercise	High	3.57	0.33	2.90	4.25	3.37	0.33	2.70	4.05
**Lipid Profile**										
Triglycerides (mg/dL)	MedDiet	Medium	78.7	12.3	54.0	103.4	147.4	12.3	122.7	172.1
	MedDiet	High	62.2	14.3	33.4	91.0	138.2	15.6	107.0	169.4
	Exercise	Medium	61.9	15.7	30.2	93.6	156.7	16.2	124.1	189.3
	Exercise	High	66.0	15.6	34.6	97.4	104.5	15.6	73.1	135.9
Total Cholesterol (mg/dL)	MedDiet	Medium	143	8.20	126.4	159.6	140	8.20	123.5	156.5
	MedDiet	High	141	9.78	120.7	160.3	119	10.14	98.6	139.4
	Exercise	Medium	165	10.92	142.5	187.5	170	11.04	147.3	192.7
	Exercise	High	167	10.59	145.4	188.6	150	10.59	128.8	171.2
LDL-Cholesterol (mg/dL)	MedDiet	Medium	74.7	5.22	64.2	85.2	78.4	5.22	67.9	88.9
	MedDiet	High	96.1	6.12	83.8	108.4	78.4	6.60	65.2	91.6
	Exercise	Medium	102.9	6.75	89.3	116.5	84.7	6.93	70.8	98.6
	Exercise	High	117.1	6.66	103.7	130.5	68.3	6.66	54.9	81.7
HDL-Cholesterol (mg/dL)	MedDiet	Medium	31.2	2.87	25.4	37.0	36.9	2.87	31.1	42.7
	MedDiet	High	26.3	3.37	19.5	33.1	21.8	3.62	14.6	29.0
	Exercise	Medium	38.1	3.72	30.6	45.6	35.4	3.81	27.7	43.1

Variable	Group	Adherence	T1 — Baseline				T2 — 6-Month Follow-up			
			Mean	SE	95%CI Low	95%CI High	Mean	SE	95%CI Low	95%CI High
	Exercise	High	32.4	3.67	25.0	39.8	28.5	3.67	21.1	35.9
Non-HDL-Cholesterol (mg/dL)	MedDiet	Medium	112.0	7.88	96.0	128.0	103.3	7.88	87.3	119.3
	MedDiet	High	114.2	9.36	95.3	133.1	97.7	9.79	78.0	117.4
	Exercise	Medium	126.5	10.43	105.3	147.7	134.3	10.58	112.9	155.7
	Exercise	High	134.4	10.15	113.9	154.9	121.8	10.15	101.2	142.4
**Cardiometabolic Risk Indices**										
TyG Index	MedDiet	Medium	7.97	0.13	7.71	8.23	8.79	0.13	8.53	9.05
	MedDiet	High	7.98	0.15	7.68	8.28	8.72	0.16	8.39	9.05
	Exercise	Medium	7.85	0.17	7.51	8.19	8.83	0.17	8.48	9.18
	Exercise	High	7.90	0.17	7.57	8.23	8.50	0.17	8.16	8.84
TG/HDL-c	MedDiet	Medium	2.54	0.52	1.49	3.59	4.33	0.52	3.28	5.38
	MedDiet	High	2.29	0.61	1.07	3.51	6.04	0.67	4.71	7.37
	Exercise	Medium	1.76	0.67	0.41	3.11	5.78	0.69	4.39	7.17
	Exercise	High	2.42	0.67	1.09	3.75	5.02	0.67	3.69	6.35
TC/HDL-c	MedDiet	Medium	4.90	0.58	3.74	6.06	4.07	0.58	2.91	5.23
	MedDiet	High	5.28	0.68	3.92	6.64	5.60	0.73	4.14	7.06
	Exercise	Medium	4.58	0.75	3.06	6.10	6.68	0.77	5.14	8.22
	Exercise	High	5.45	0.74	3.96	6.94	6.96	0.74	5.48	8.44
**Hepatic Biomarkers**										
Total Bilirubin (mg/dL)	MedDiet	Medium	2.09	0.20	1.7	2.5	1.48	0.200	1.1	1.9
	MedDiet	High	2.08	0.23	1.6	2.5	1.14	0.257	0.6	1.7
	Exercise	Medium	1.81	0.25	1.3	2.3	1.31	0.257	0.8	1.8
	Exercise	High	1.81	0.25	1.3	2.3	1.11	0.253	0.6	1.6
AST (U/L)	MedDiet	Medium	28.2	2.14	23.9	32.5	29.0	2.14	24.7	33.3
	MedDiet	High	24.6	2.49	19.6	29.6	24.1	2.71	18.7	29.5
	Exercise	Medium	34.6	2.74	29.0	40.2	26.5	2.82	20.8	32.2
	Exercise	High	26.3	2.72	20.9	31.7	27.5	2.72	22.1	32.9
ALT (U/L)	MedDiet	Medium	16.1	4.54	6.9	25.3	22.7	4.54	13.5	31.9
	MedDiet	High	33.1	5.40	22.1	44.1	26.4	5.65	15.0	37.8

Variable	Group	Adherence	T1 — Baseline				T2 — 6-Month Follow-up			
			Mean	SE	95%CI Low	95%CI High	Mean	SE	95%CI Low	95%CI High
	Exercise	Medium	25.3	6.01	13.1	37.5	26.5	6.10	14.1	38.9
	Exercise	High	20.1	5.85	8.2	32.0	23.7	5.85	11.8	35.6
GGT (U/L)	MedDiet	Medium	11.2	1.15	8.9	13.5	14.8	1.15	12.4	17.2
	MedDiet	High	14.7	1.37	11.9	17.5	15.5	1.44	12.6	18.4
	Exercise	Medium	14.9	1.52	11.9	17.9	16.1	1.54	12.9	19.3
	Exercise	High	11.8	1.48	8.8	14.8	12.9	1.48	9.9	15.9

Note. Estimated marginal means (EMMs) from the linear mixed model (REML), adjusted for zBMI/A and zMETs at their grand mean. Grey band (T1): baseline; teal band (T2): 6-month follow-up. SE = standard error. 95% CI computed using the Wald method with Satterthwaite-approximated df. Negative CI lower bounds reflect sampling uncertainty and do not imply physiologically impossible values. Medium = KIDMED 4–7; High = KIDMED ≥ 8.

Supplementary Table S5. *FDR-adjusted (Benjamini–Hochberg) p-values for all fixed effects and interactions across outcome families.*

Variable	T	G	A	T×G	T×A	G×A	T×G×A	G(Δ)	A(Δ)	G×A(Δ)	G(Δ%)	A(Δ%)	G×A(Δ%)
	crude / FDR	*crude / FDR*	*crude / FDR*	*crude / FDR*	*crude / FDR*	*crude / FDR*	*crude / FDR*	*crude / FDR*	*crude / FDR*	*crude / FDR*	*crude / FDR*	*crude / FDR*	*crude / FDR*
***Domain:** Glycemic Control*													
Glucose	.519 / .710	.735 / .840	.159 / .302	.724 / .840	.363 / .524	.563 / .732	.783 / .847	.642 / .795	.355 / .522	.826 / .871	.496 / .703	.519 / .710	.955 / .959
Insulin	<.001 / <.001	.913 / .937	.011 / .031	<.001 / .004	.024 / .061	.531 / .714	.022 / .058	.009 / .025	.063 / .135	.091 / .182	<.001 / <.001	<.001 / .002	.268 / .427
HOMA-IR	.001 / .004	.771 / .847	.005 / .014	.016 / .042	.033 / .075	.593 / .751	.174 / .316	.026 / .063	.070 / .144	.346 / .519	<.001 / <.001	<.001 / <.001	.776 / .847
HOMA-β	<.001 / <.001	.737 / .840	.032 / .075	.068 / .143	.686 / .823	.597 / .751	.060 / .134	.216 / .383	.854 / .888	.307 / .479	<.001 / .002	.228 / .395	.163 / .303
QUICKI	<.001 / <.001	.248 / .412	.001 / .004	<.001 / .002	<.001 / <.001	.246 / .412	.261 / .424	<.001 / <.001	<.001 / <.001	.959 / .959	<.001 / <.001	<.001 / <.001	.743 / .840
iMcA	<.001 / <.001	.334 / .511	.004 / .013	<.001 / <.001	<.001 / <.001	.146 / .285	.543 / .718	<.001 / .001	<.001 / .004	.793 / .847	.004 / .011	<.001 / .001	.662 / .807
***Domain:** SPINA-Carb*													
SPINA-Gβ	<.001 / <.001	.815 / .815	.021 / .054	.004 / .017	.610 / .661	.590 / .657	.020 / .054	.027 / .066	.563 / .646	.177 / .345	<.001 / <.001	.018 / .054	.336 / .510
SPINA-GR	.017 / .054	.805 / .815	.042 / .091	.100 / .205	.255 / .427	.721 / .760	.263 / .427	.516 / .610	.369 / .514	.513 / .610	.251 / .427	.394 / .529	.353 / .510
SPINA-DI	<.001 / <.001	.453 / .570	.002 / .011	<.001 / <.001	.005 / .020	.407 / .529	.031 / .071	<.001 / <.001	.008 / .031	.345 / .510	<.001 / <.001	.018 / .054	.241 / .427
***Domain:** Lipid Profile*													
Triglycerides	<.001 / <.001	.539 / .631	.140 / .257	.693 / .751	.096 / .205	.651 / .717	.031 / .096	.570 / .650	.143 / .257	.112 / .221	.242 / .366	.111 / .221	.838 / .865
Total Cholesterol	.002 / .016	.021 / .079	.271 / .390	.265 / .390	<.001 / .008	.863 / .876	.783 / .834	.042 / .107	.003 / .019	.614 / .688	.015 / .069	.002 / .016	.470 / .576
LDL-Cholesterol	<.001 / <.001	.094 / .205	.374 / .477	<.001 / <.001	<.001 / <.001	.276 / .390	.412 / .515	.005 / .032	.001 / .011	.544 / .631	.025 / .086	.036 / .101	.322 / .436
HDL-Cholesterol	.369 / .477	.222 / .357	.010 / .056	.193 / .330	.059 / .141	.541 / .631	.137 / .257	.224 / .357	.146 / .257	.087 / .202	.235 / .364	.013 / .065	.043 / .107
Non-HDL-Cholesterol	.016 / .069	.042 / .107	.819 / .859	.098 / .205	.022 / .079	.969 / .969	.303 / .419	.013 / .065	.034 / .101	.345 / .458	.017 / .069	.030 / .096	.225 / .357
***Domain:** Cardiometabolic Risk*													
TyG Index	<.001 / <.001	.555 / .833	.529 / .833	.967 / >.999	.084 / .364	.687 / .837	.283 / .649	.670 / .837	.148 / .412	.430 / .833	.617 / .837	.143 / .412	.598 / .837
TG/HDL-c	<.001 / <.001	.929 / >.999	.510 / .833	.385 / .833	.666 / .837	.459 / .833	.008 / .050	.555 / .833	.847 / .964	.029 / .141	.550 / .833	.997 / >.999	.453 / .833
TC/HDL-c	.009 / .050	.204 / .509	.209 / .509	<.001 / .008	.637 / .837	.751 / .887	.140 / .412	.002 / .016	.865 / .964	.134 / .412	.002 / .016	>.999 / >.999	.129 / .412
***Domain:** Hepatic Biomarkers*													
Total Bilirubin	<.001 / .002	.369 / .659	.419 / .660	.577 / .769	.406 / .659	.832 / .918	.844 / .918	.044 / .236	<.001 / .002	.231 / .572	.276 / .615	<.001 / .004	.284 / .615
AST	.187 / .486	.399 / .659	.076 / .329	.141 / .485	.106 / .424	.883 / .918	.035 / .236	.494 / .722	.345 / .659	.491 / .722	.361 / .659	.683 / .866	.782 / .918
ALT	.514 / .722	.911 / .929	.529 / .724	.506 / .722	.132 / .485	.158 / .485	.030 / .236	.877 / .918	.296 / .616	.047 / .236	.805 / .918	.813 / .918	.035 / .236
GGT	.001 / .013	.940 / .940	.673 / .866	.309 / .618	.178 / .486	.043 / .236	.168 / .485	.277 / .615	.380 / .659	.162 / .485	.050 / .236	.849 / .918	.873 / .918

Note. Each cell shows crude p-value / FDR-adjusted p-value (Benjamini–Hochberg) within biologically defined outcome families. **Effects** — T: Time; G: Group (MedDiet vs. Exercise); A: Adherence (Medium vs. High); T×G, T×A, G×A: two-way interactions; T×G×A: three-way interaction; G(Δ), A(Δ), G×A(Δ): GLM effects on absolute change; G(Δ%), A(Δ%), G×A(Δ%): GLM effects on percentage change. P-values shown in bold dark red indicate statistical significance ($p < .05$). Grey italic values indicate non-significance. All models adjusted for zBMI/A and zMETs as covariates. **Abbreviations:** iMcA = McAuley index; SPINA-Gβ = β-cell secretory capacity; SPINA-GR = insulin receptor sensitivity; SPINA-DI = disposition index; TyG = triglyceride–glucose index; TG/HDL-c = triglyceride-to-HDL cholesterol ratio; TC/HDL-c = total cholesterol-to-HDL ratio; AST = aspartate aminotransferase; ALT = alanine aminotransferase; GGT = gamma-glutamyl transferase; Medium = KIDMED 4–7; High = KIDMED ≥ 8.
